# Obesity- and tumor-derived signals drive cancer-associated state transitions in breast mesenchymal stromal/stem cells reprogrammed by IL1RA or JAK inhibition

**DOI:** 10.1186/s40164-026-00747-7

**Published:** 2026-02-05

**Authors:** Andreas Ritter, Samira Catharina Hoock, Nina-Naomi Kreis, Susanne Roth, Rosario Carolina Torres Colin, Alexandra Friemel, Julia Maria Wildner, Ilona Scherr, Frank Louwen, Christine Solbach, Juping Yuan

**Affiliations:** Obstetrics and Prenatal Medicine, Gynecology and Obstetrics, University Hospital Frankfurt, J. W. Goethe-University, Theodor-Stern-Kai 7, 60590 Frankfurt, Germany

**Keywords:** Obesity, Breast cancer, Stromal cell plasticity, Cancer-associated fibroblasts, Adipose tissue-derived mesenchymal stromal/Stem cells, TGFβ signaling, IL1/JAK pathway, Cancer stem cells, Epithelial-to-mesenchymal transition

## Abstract

The tumor microenvironment (TME) in breast cancer is shaped by reciprocal interactions between cancer cells and their surrounding stromal populations. Here, we show that breast adipose tissue-derived stromal/stem cells (bASCs) undergo distinct state transitions in response to tumor cues and systemic metabolic status. Using primary bASCs derived from tumor-adjacent and tumor-distant adipose tissues of breast cancer patients with or without obesity, we identify two functionally distinct, tumor-educated stromal phenotypes: a cytokine-rich inflammatory CAF-like (iCAF) state predominating in lean-adjacent bASCs (ln-aT), and a myofibroblastic CAF-like (myCAF) state emerging in obese-adjacent bASCs (ob-aT). Importantly, transforming growth factor β (TGFβ) is sufficient to induce myCAF-like reprogramming in obesity-primed bASCs, while interleukin 1 (IL1)-Janus kinase (JAK) signaling promotes iCAF features. Re-analysis of single-cell RNA-seq data of breast cancer samples reveals an increased TGFβ expression across stromal and immune cell types in individuals with obesity. Mechanistically, IL1 receptor blockade (anakinra) or JAK inhibition (AZD1480) reverses both iCAF and myCAF phenotypes and functionally suppresses stromal-driven epithelial-mesenchymal transition as well as cancer stemness in breast cancer cells. These findings establish a mechanistic link between obese cues, stromal plasticity, and breast cancer progression, and reveal IL1/JAK signaling as a tractable axis to therapeutically reprogram the breast cancer stroma.

**Graphical Abstract:**

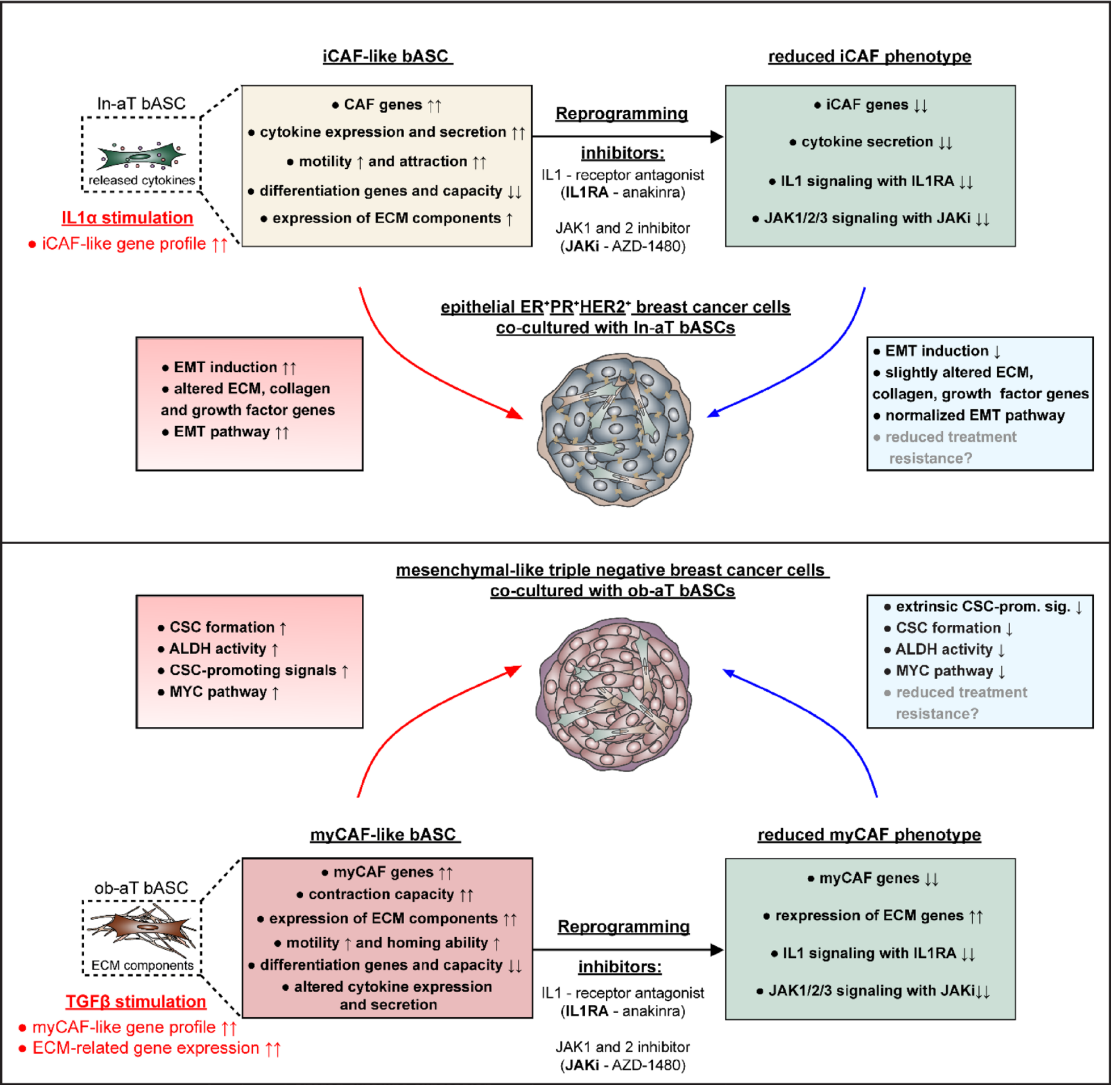

**Supplementary Information:**

The online version contains supplementary material available at 10.1186/s40164-026-00747-7.

## Introduction

Breast cancer is the most prevalent malignancy among women worldwide, accounting for about 32% of all female cancers [1]. Globally, its burden continues to rise, with 2.08 million new cases and 0.66 million deaths reported in 2021 [2]. It is a heterogeneous disease with highly variable survival outcomes influenced by tumor biology, therapeutic regimens, hormonal and menopausal status, as well as socioeconomic determinants of health, including obesity, defined by a body mass index (BMI ≥ 30) [3]. According to the expression levels of estrogen receptor (ER), progesterone receptor (PR), and human epidermal growth factor receptor 2 (HER2), breast cancer can be divided into four molecular subtypes: luminal A, luminal B, HER2-positive (HER2^+^), and triple-negative breast cancer (TNBC) [4]. The TNBC subtype accounts for 15–20% of all breast cancer cases and is characterized by the absence of receptor expression on the cell surface. This feature makes TNBC the most lethal subtype, as it is largely resistant to current targeted therapies [5, 6].

The relationship between obesity and breast cancer risk and aggressiveness is multifaceted. In premenopausal patients, obesity was identified as a significant protective factor against breast cancer development [7]. However, clinical studies have also shown a link between TNBC and obesity in premenopausal patients [8, 9]. Moreover, in postmenopausal women, obesity is well known to be a main risk factor for breast cancer development [10, 11]. In this group, tumors tend to be larger, exhibit greater resistance to hormone therapy, and have a higher incidence of metastasis [11]. These findings have been attributed to the substantially increased production of estrogen, and leptin from adipose tissue in postmenopausal patients with obesity [12]. The molecular connection between obesity and breast cancer development is only partially understood, involving complex cellular and molecular mechanisms that extend beyond the simple production of adipose tissue-related hormones and adipokines.

In severe obesity (BMI ≥ 35), adipose tissue is marked by adipocyte hypertrophy, cellular senescence, inflammation, increased secretion of cytokines/adipokines, hypoxia, stress, extracellular matrix (ECM) remodeling, and fibrosis [13]. Hypertrophy and impaired vascularization reduce oxygen supply, activating hypoxia-inducible factor (HIF) pathways and exacerbating fibrosis, and tissue dysfunction [14]. These changes, particularly hypoxia and fibrosis, further foster immune cell recruitment and activation, especially macrophages and T cells. Moreover, hypoxic tumors promote the recruitment of regulatory T cells through CCL28 (C-C motif chemokine ligand 28), which in turn suppresses effector T-cell function [15], prompting adipose tissue to markedly upregulate the secretion of pro-inflammatory cytokines [13, 16, 17]. The elevated cytokines from deregulated macrophages, T cells, and neutrophils impair adipogenic differentiation and reinforce metabolically induced meta-inflammation [17, 18].

In line with these alterations, dysfunctional adipocytes, obesity-associated pathologies, and elevated estrogen levels are recognized factors that directly contribute to the development and progression of breast cancer and remodeling in its tumor microenvironment (TME) [16, 19]. Central components of the TME in primary and metastatic tumors include cancer-associated fibroblasts (CAFs) [20, 21], tumor-associated macrophages (TAM) [22], mal-differentiated CD4^+^ T cells [23], tumor-infiltrating lymphocytes [24], and breast adipose tissue-derived mesenchymal stromal/stem cells (bASCs) [25, 26]. They contribute to various aspects of tumor biology, including cell proliferation, migration, invasion, metastasis, and therapy resistance, and thereby play crucial roles in supporting tumorigenesis [27]. Within the TME of breast cancer, at least three major subtypes of CAFs have been identified: myofibroblastic CAFs (myCAFs), inflammatory CAFs (iCAFs), and antigen-presenting CAFs (apCAFs) [27]. These subtypes exhibit distinct functional roles in tumorigenesis with key features, like ECM remodeling by myCAFs, secretion of inflammatory cytokines by iCAFs, and expression of MHCII (major histocompatibility complex II) by apCAFs [27]. Reflecting the high plasticity of these cell types and the dramatic impact of obesity, our recent study demonstrates that breast cancer patients with obesity exhibit an increased population of myCAF-like bASCs within the TME. In contrast, lean patients predominantly display iCAF-like bASCs [25]. This differential prevalence results in fundamentally distinct cell-cell interactions, significantly influencing epithelial-to-mesenchymal transition (EMT), cancer stem cell (CSC) formation, and chemotherapy resistance in breast cancer cell spheroids [25].

CAFs are widely recognized across various tumor entities, including breast cancer, for their strong association with malignant progression and poor prognosis [20, 28]. Recently, Croizer et al. applied spatial transcriptomics and trajectory analysis to map iCAF-myCAF plasticity in breast cancer [20]. They identified multiple CAF subtypes and their intercellular interaction networks, revealing transitions from the inflammatory (detox-iCAF) state to ECM-producing myCAFs [20], though functional validation of these subtypes remains pending. Inhibition of IL1 (interleukin-1) signaling with its receptor antagonist (IL1RA) anakinra mitigated therapy resistance, modulated immune cell dynamics, and decreased the iCAF phenotype within the TME in colorectal and pancreatic cancer [29, 30]. On the other hand, blockade of the JAK1/2 (Janus kinase 1/2) pathway with the inhibitor (AZD1480) effectively attenuated iCAF functions in pancreatic ductal adenocarcinoma. In addition, treatment with TGFβ suppressed iCAF cytokine secretion by downregulating IL1R1 and promoting transdifferentiation into myCAFs [31].

To detail the reciprocal interactions between the TME, breast cancer cells, and bASCs, we have performed multiple functional assays, in combination with analysis of RNA-seq results, and reanalysis of single-cell RNA-seq (scRNA-seq) data (BioKey, NCT03197389). Our findings demonstrate that cancer-educated bASCs exhibit significantly upregulated expression of ECM-related genes and proteins, as well as elevated motility. Furthermore, multiple cell types show increased TGFβ gene expression, and treatment with TGFβ is sufficient to induce a robust myCAF-like phenotype in ob-dT bASCs. Of importance, treatment with the JAK1/2 inhibitor AZD1480 or IL1RA anakinra partially reprogrammed these cell phenotypes, reducing EMT induction by treated ln-aT bASCs and decreasing CSC formation by treated ob-aT bASCs through the downregulation of MYC-signaling in TNBC cells.

## Materials and methods

### bASC isolation, cell lines, cell culture, and differentiation

This study was approved by the Ethics Committee of Goethe University Hospital Frankfurt (reference No: 443/11 and 4/09) with informed consent from all donors. Clinical information of breast cancer patients (*n* = 34) enrolled in this study is summarized in Table S1. Isolated bASCs from four patients treated with tamoxifen, aromatase inhibitors, or radiotherapy were used for some functional assays but not included in the RNA-seq experiments. Collected samples during surgery included mammary adipose tissues, which were ≥ 9 cm distant from the tumor (dT) and ≤ 3 cm adjacent (aT) to the tumor. All bASCs were isolated from adipose tissues following a modified protocol [32]. Tissues were washed, minced, digested with collagenase, neutralized with medium, centrifuged, filtered, and treated with red blood cell lysis buffer (155 mM NH_4_Cl, 10 mM KHCO_3_, and 0.1 mM EDTA). Isolated cells were cultured in DMEM with 20% FBS and antibiotics (100 g/ml streptomycin, 100 U/ml penicillin), and non-adherent cells were removed. Early passages (P2-P6) of bASCs were used for experiments. Both ln-bASCs and ob-bASCs were characterized (Tables S2 and S3). The breast cancer cell lines MCF7^(ER+, PR+, HER2+low)^ and MDA-MB-231^(ER−, PR−, HER2−)^ were chosen based on the status of their surface receptors, namely ER, PR and HER2 [33], and their low (MCF7)- and high metastatic potential (MDA-MB-231). These cells were cultured as instructed (ATCC, Wesel).

### Cell cycle distribution, flow cytometry, and aldehyde dehydrogenase (ALDH) activity assay

Cell cycle distribution and cell surface marker measurement of bASCs via fluorescence-activated cell sorting (FACS) are detailed in supplementary information. ALDH activity was assessed using the ALDEFLUOR kit (Stem Cell Technologies, Cat# 01700) following the manufacturer’s protocol. Briefly, bACSs were treated with DMSO, IL1RA (10 µg/ml [30]), or JAKi (500 nM [31]) for 72 h and subsequently co-cultured with MDA-MB-231 cells for up to 14 days. Cells were then incubated with BODIPY-aminoacetaldehyde (BAAA), which is metabolized to BODIPY-aminoacetate. This conversion leads to fluorescence accumulation in cells with active ALDH. FACSCalibur™ (BD Bioscience, Heidelberg) was used to classify cells as ALDH-bright (ALDH^br^), ALDH-low (ALDH^low^), or ALDH-negative. Data were analyzed using Flowing software (version 2.5.1, Turku Bioimaging).

### Indirect Immunofluorescence staining, image acquisition, and signal intensity measurement

Indirect immunofluorescence staining, image acquisition, and signal intensity quantification are detailed in supplementary information.

### RNA extraction, real-time quantitative PCR (qPCR), and RNA sequencing (RNA-seq)

RNA extraction, qPCR, probes, and RNA-seq are detailed in supplementary information.

### Enzyme-linked immunosorbent assay (ELISA)

Cytokine quantifications via ELISA are detailed in supplementary information.

### EMT evaluation

EMT assessments are described in supplementary information and as reported [25].

### Motility assay and homing assay

The motility and homing assays are described in supplementary information and were evaluated, as reported [34].

### Collagen gel contraction assay

The collagen gel contraction assay was performed as described [35]. 50,000 bASCs were resuspended in 300 µl of reduced medium supplemented with 160 µl of 3 mg/ml rat tail collagen I (#A1048301; Thermo Fisher Scientific, Waltham, MA), 16.8 µl of 10 × PBS, and 8 µl of 1 mol/l sodium hydroxide. The resulting suspension was transferred into a 24-well plate and allowed to solidify at 37 °C with 5% CO₂ for 45 min. Following solidification, the collagen disks were released and imaged after 24 h incubation.

Collagen gel contraction was documented using a AxioObserver Z1 microscope equipped with an AxioCam MRm camera (Zeiss), and the circumference of the collagen disks was measured using ImageJ software. The contraction index was calculated as follows: $\:contraction\:index=\:100\mathrm{*}\left(\frac{circumference\:at\:time\:point}{initial\:circumference}\right)$, and is reported as percent (%).

### scRNA-seq analysis

Raw gene expression matrices and cell clustering analyses were initially performed by Bassez and Vos et al. using the Seurat v3 R package [36]. The resulting Seurat objects, which included raw data, cluster assignments, and annotations for cell types and subtypes, were obtained and further processed using Seurat version 4.1.1. Nguyen et al. included eight cell types in their analyses [37]: cancer cells, B cells, T cells, macrophages/monocytes, dendritic cells, mast cells, fibroblasts, and endothelial cells. Differentially expressed genes (DEGs) were defined as those with an absolute average log₂ fold change (|avg_logFC|) ≥ 0.5 and a p-value < 0.05. The absolute value was used solely as a selection threshold to capture both upregulated and downregulated genes. For visualization in the heatmap, we plotted the signed avg logFC values to indicate directionality (positive values = upregulated in obese; negative values = upregulated in lean). In parallel, single-cell RNA sequencing data from GSE195665 [38] were reprocessed using Seurat v5. Cells from six patients with normal weight (BMI < 25) and four patients with obesity (BMI ≥ 30) were included for BMI-stratified analysis (patient metadata available in Supplementary Material 1). Raw gene expression counts were normalized using a log-normalization approach, where feature counts for each cell were scaled by total expression, multiplied by a scaling factor (10,000), and log-transformed. Immune cell populations were annotated using SingleR based on reference transcriptome data. For visualization of gene expression, log-normalized values were further z-score-transformed across all cells, yielding scaled expression values. These standardized values were used to generate violin plots, enabling comparison of relative gene expression patterns across BMI categories within defined immune cell subsets.

### Gene set enrichment analysis

Gene Set Enrichment Analysis (GSEA) was conducted using the GSEA software developed by the Broad Institute [39]. RNA-seq differential expression results, including gene identifiers and corresponding expression statistics, were used as input. Genes were ranked according to their expression changes between conditions. The GSEA tool was run with default parameters, using the Molecular Signatures Database (MSigDB) hallmark gene sets to identify significantly enriched pathways. Specific gene enrichment profiles were pre-defined detailed in Supplementary Material 2. Enrichment significance was determined based on the normalized enrichment score (NES), nominal (NOM) p-values and false discovery rate (FDR) q-values, with thresholds set at *p* < 0.05 and FDR q < 0.25.

### Statistical analysis

An outlier test was performed with all data sets prior to statistical analysis. Student’s t-test (two-tailed and paired or homoscedastic) was used to evaluate the significance of the difference between diverse groups for gene expression analysis and cell cycle distribution. The statistical evaluation of the single-cell tracking assay and immunofluorescence quantification was performed by using an unpaired Mann-Whitney U test (two-tailed). The difference is considered statistically significant, when *p* < 0.05.

## Results

### Key signaling pathways are altered in breast cancer-associated bASCs

To reveal the impacts of breast cancer and obesity on global gene expression, transcriptomic analysis was performed in ln- and ob-aT bASCs (ln, lean; ob, obese; aT, adjacent to the tumor) compared to their corresponding control dT bASCs (dT, distant from the tumor). Clinical data of the patients are summarized in Table S1. All bASCs used in this study were characterized using positive cell surface markers cluster of differentiation 73 (CD73), CD90, CD146 and negative markers CD14, CD31, and CD106 (Tables S2 and S3). Total RNA was extracted from each sample of all four bASC subgroups for whole-genome RNA-seq analysis. A part of the data was previously reported [25], and the data were merged and re-analyzed in this study as indicated. Differential expression analysis revealed substantial transcriptomic differences among the groups. A total of 1679 and 1342 genes were significantly deregulated in ln-aT versus ln-dT, and ob-aT versus ob-dT bASCs, respectively (Fig. S1A and B). Comparisons between ln-aT and ob-aT, as well as ln-dT and ob-dT bASCs, revealed over 2500 and 2600 differentially expressed genes (DEGs), respectively (Fig. S1C and D), reflecting the impact of breast cancer as well as metabolic status. The “Kyoto Encyclopedia of Genes and Genomes” (KEGG) pathway enrichment analysis of DEGs between ln-aT and ob-aT bASCs highlighted significant alterations in transcriptional regulatory pathways, including TGFβ signaling, actin cytoskeleton regulation, and insulin signaling/resistance (Fig. S1E). A Venn diagram analysis further illustrated these differences, showing a larger set of uniquely deregulated genes in ln-aT bASCs (542 genes) compared to ob-aT bASCs (217 genes) (Fig. S1F). Notably, ln-aT bASCs exhibited pronounced upregulation of genes involved in de-differentiation and cancer-associated pathways, such as TGFβ signaling (5 genes), nuclear factor kappa B (NF-ĸB) pathway (12 genes), mitogen-activated protein kinase (MAPK) signaling pathway (19 genes), and hedgehog (Hh) signaling pathway (6 genes) (Fig. S1G). On the other hand, ob-aT bASCs displayed deregulation of pathways associated with cell adhesion (15 genes), metabolic processes (60 genes), phosphoinositide-3-kinase–Akt serine/threonine kinase (PI3K–Akt) signaling pathway (18 genes), and MAPK signaling (21 genes) (Fig. S1H), which have been linked to the emergence of myCAF-like phenotypes [40]. Gene Ontology (GO) analysis further revealed enrichment of genes related to the extracellular region, extracellular space, cell motility, and cytoskeletal protein binding in both ln-aT and ob-aT bASCs (Fig. S1I and J), indicating a functional transition characteristic of tumor- as well as obesity-educated stromal cells. These findings underscore the tumor- and TME-driven reprogramming of bASCs, showing activation of tumor-promoting pathways (TGFβ, NF-κB, MAPK, Hh) in ln-aT bASCs, and manifestation of myCAF-like features enriched in cell adhesion, metabolic, and PI3K-Akt signaling in ob-aT bASCs, supported by previous studies [41–43].

### myCAF-like bASCs express high levels of collagens and fibronectin to modulate the ECM

**Fig. 1:**
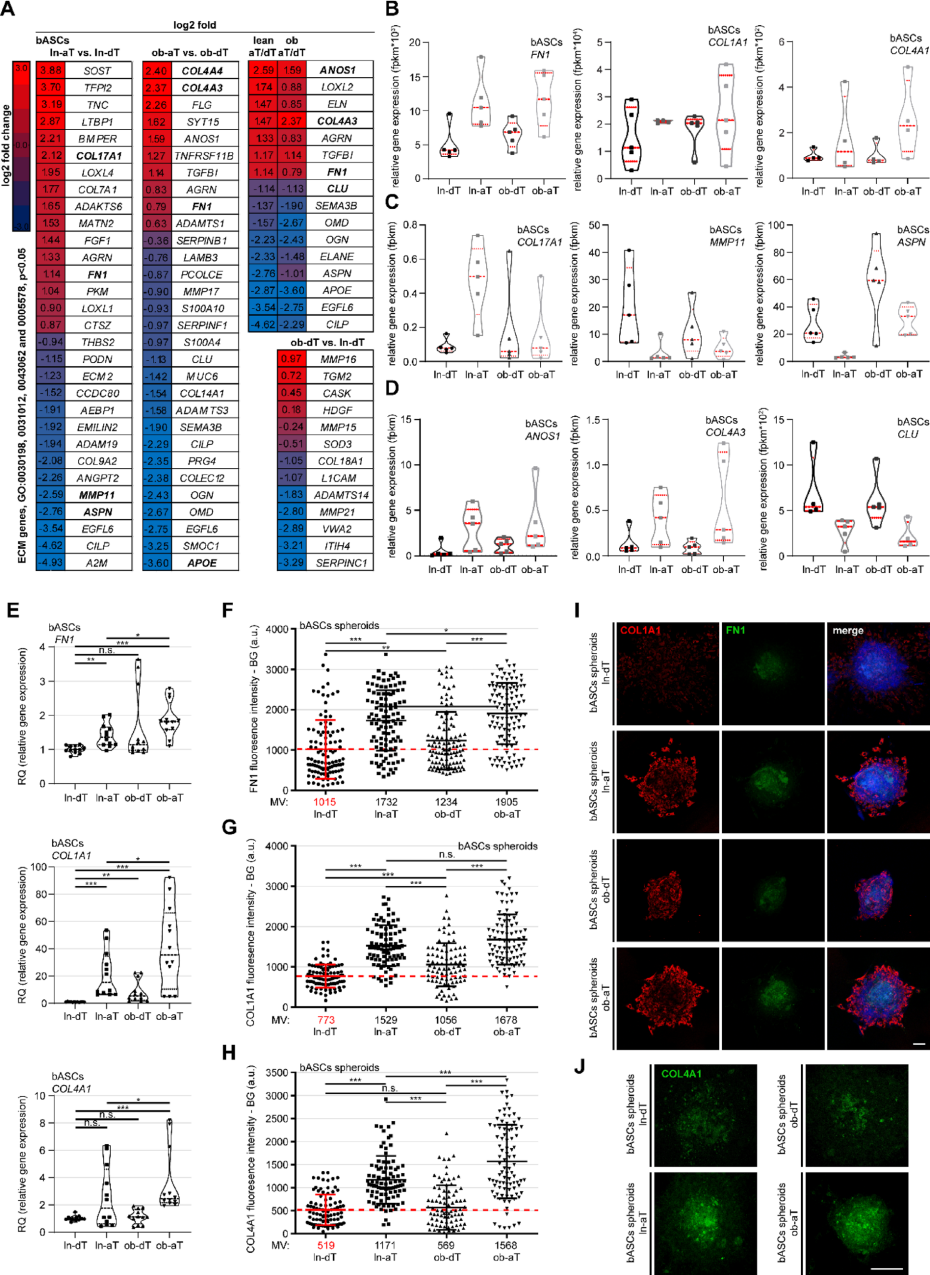
MyCAF-like bASCs drive ECM remodeling through elevated collagen and fibronectin expression. **A**–**D** Total RNAs were extracted from each sample of bASC subgroups (ln-dT, ln-aT, ob-dT, and ob-aT, 5 samples for each subgroup) for RNA-seq analysis. **A** Heatmaps illustrating differentially expressed genes (DEGs) in ECM-related pathways (GO: 0030198, 0031012, 0043062, and 0005578). Comparisons include ln-aT vs. ln-dT (1st panel), ob-aT vs. ob-dT (2nd panel), shared DEGs between ln-aT/dT and ob-aT/dT (3rd panel), and ob-dT vs. ln-dT (4th panel). Log2-fold changes are represented by color intensity (red: + 3; dark blue: − 3). **B**–**D** Violin plots showing relative gene expression levels (fragments per kilobase per million mapped fragments (fpkm)) of selected ECM-related DEGs. **B** DEGs in ob-aT bASCs, **C** DEGs in ln-aT bASCs, and **D** DEGs shared by ln-aT/dT and ob-aT/dT subgroups. **E** Quantitative PCR (qPCR) analysis of ECM-related genes *FN1*, *COL1A1*, and *COL4A1* in bASC subgroups (ln-dT, ln-aT, ob-dT, and ob-aT). Results are presented as RQ ± SEM from three independent experiments. **F**–**H** Mean fluorescence intensity (MFI) of FN1, COL1A1, and COL4A1 in 3D bASC spheroids, shown as scatter plots. Data are from three independent experiments (*n* = 3, with 90 spheroids per condition and subgroup) and represent mean ± SEM. **I** and **J** Representative immunofluorescence images of 3D bASC spheroids stained for ECM proteins. **I** FN1 (green), COL1A1 (red), and DNA (DAPI, blue). **J** COL4A1 (green). Scale bars, 25 μm. An unpaired Mann-Whitney U test was used in **F**–**H**. Student’s t-test was used in **E**. ∗*p* < 0.05, ∗∗*p* < 0.01, ∗∗∗*p* < 0.001

As remodeling of the ECM is a hallmark of cancer-educated cells, particularly those resembling myCAF-like cells [44], analysis was focused on ECM-related gene ontology pathways, including “ECM”, “extracellular matrix organization,” “extracellular structure organization,” and “proteinaceous extracellular matrix”. In fact, ln-aT bASCs exhibited a multifaceted modulation of ECM-related genes, with an upregulation of collagen type I alpha 1 chain (*COL1A1)*, *COL4A1*, *COL17A1*, and fibronectin 1 (*FN1)*, and a downregulation of matrix metallopeptidase 11 (*MMP11)*, asporin (*ASPN)*, and *COL9A2* (Fig. 1A–C, left heatmap), genes implicated in fibrotic processes [45, 46], whereas ob-aT bASCs displayed a myCAF-specific profile, including upregulated *COL1A1*, *COL4A1*, *COL4A3*, *COL4A4*, and *FN1*, along with downregulated apolipoprotein E (*APOE)* (Fig. 1A, B, D; middle heatmap), an indirect TGFβ pathway modulator [47]. Comparative analysis of ln- and ob-aT bASCs revealed minimal overlap in ECM-related gene expression, with *FN1*, *COL4A3*, and anosmin 1 (*ANOS1)* elevated in both types (Fig. 1A, right upper heatmap), underscoring their common yet distinct cancer-educated phenotypes. Notably, their controls ln-dT versus ob-dT bASCs showed no fibrosis-associated gene profile (Fig. 1A, right lower heatmap).

Validation via qPCR supported the results from RNA-seq, showing a significantly higher expression of *FN1*, *COL1A1*, and *COL4A1* in both aT bASC subtypes, with enhanced levels in ob-aT bASCs relative to ln-aT bASCs (Fig. 1E). To mimic the physiological situation, bASCs were formed into 3D spheroids, which were fixed and stained with antibodies against COL1A1 and FN1 (Fig. 1I), or COL4A1 (Fig. 1J) for protein intensity evaluation. The relative quantification of fluorescence intensity verified the notion that both aT bASC subtypes increased the expression of FN1, COL1A1, and COL4A1, but ob-aT bASCs displayed the highest levels of the protein expression compared to other subtypes (Fig. 1F–J).

In sum, both cancer-educated aT bASC subtypes have an enhanced expression of ECM-related genes, favoring fibrosis and ECM stiffness. Moreover, ob-aT bASCs, which showed an increased population of myCAF-like cells [16, 25], had the highest gene and protein expression of key ECM remodeling factors.

### The motility capacity of ln- and ob-aT bASCs is increased via different molecular mechanisms

Cell motility is an important functional hallmark of ASCs [17, 48]. To characterize this aspect, we examined RNA-seq data related to cell motility (GO:0048870) and focal adhesion (GO:0005925) pathways. The examination revealed two distinct modes of motility enhancement in lean and obese bASC subgroups. The ln-aT bASCs showed increased motility, potentially driven by the upregulation of various motility promoting chemokines and cytokines, including C-X-C motif chemokine ligand 1 (*CXCL1)*, *CXCL3*, *CXCL5*, *CXCL6*, *CXCL8*, *CXCL11*, *CCL5*, and *IL1β* (Fig. S2A, left heatmap, and B). In contrast, ob-aT bASCs exhibited limited chemokine/cytokine modulation, instead showing upregulated cytoskeletal components, such as tektin 2 (*TEKT2)*, associated with microtubule stability [49], actin beta like 2 (*ACTBL2)*, essential for actin-based motility [50], and integrin subunit beta 2 (*ITGB2)*, mediating cell adhesion via intercellular adhesion molecule 1 (ICAM-1) binding [51] (Fig. S2A, middle heatmap, C and D).

Genes deregulated in both ln- and ob-aT bASCs including *ACTBL2* and *ITGB2*, with significantly elevated expression in ob-aT bASCs (Fig. S2A, right upper heatmap and D). Additionally, *FOXE1*, encoding a transcription factor involved in migration and invasion [52], was notably upregulated in both bASC subtypes (Fig. S2A, right upper heatmap). Interestingly, stromal cells were reported to typically exhibit obesity-induced activation markers in mice [53, 54]. However, comparative analyses of ln- and ob-dT bASCs suggested a prominent downregulation of fibroblast activation protein alpha (*FAP)* and SMAD family member 4 (*SMAD4)*, key genes for activated stroma/fibroblasts, in obese cells (Fig. S2A, right lower heatmap).

To underscore the findings in gene expression, motility and homing attraction assays were performed. While ob-dT bASCs demonstrated the lowest motility (Fig. S2E–H) and homing potential (Fig. S2I–K), ln-aT bASCs displayed the highest motility rate (Fig. S2E–H), likely due to enhanced cytokine secretion and signaling processing. Consistent with this hypothesis, ln-aT bASCs showed markedly increased homing attraction at 12 h and 16 h towards MDA-MB-231 cells, indicating their robust cytokine-dependent signaling compared to other subtypes (Fig. S2I–K). Ob-aT bASCs also exhibited elevated homing capacity, although it was significantly lower than that of ln-aT bASCs at 16 h (Fig. S2I–K).

These data demonstrate that ln- and ob-aT bASCs exhibit distinct motility mechanisms: the motility of ln-aT bASCs is predominantly driven via cytokine upregulation and action, while ob-aT bASC motility relies mostly on cytoskeletal and adhesion molecules.

### bASCs de-differentiate into distinct CAF-like phenotypes with functional changes

**Fig. 2:**
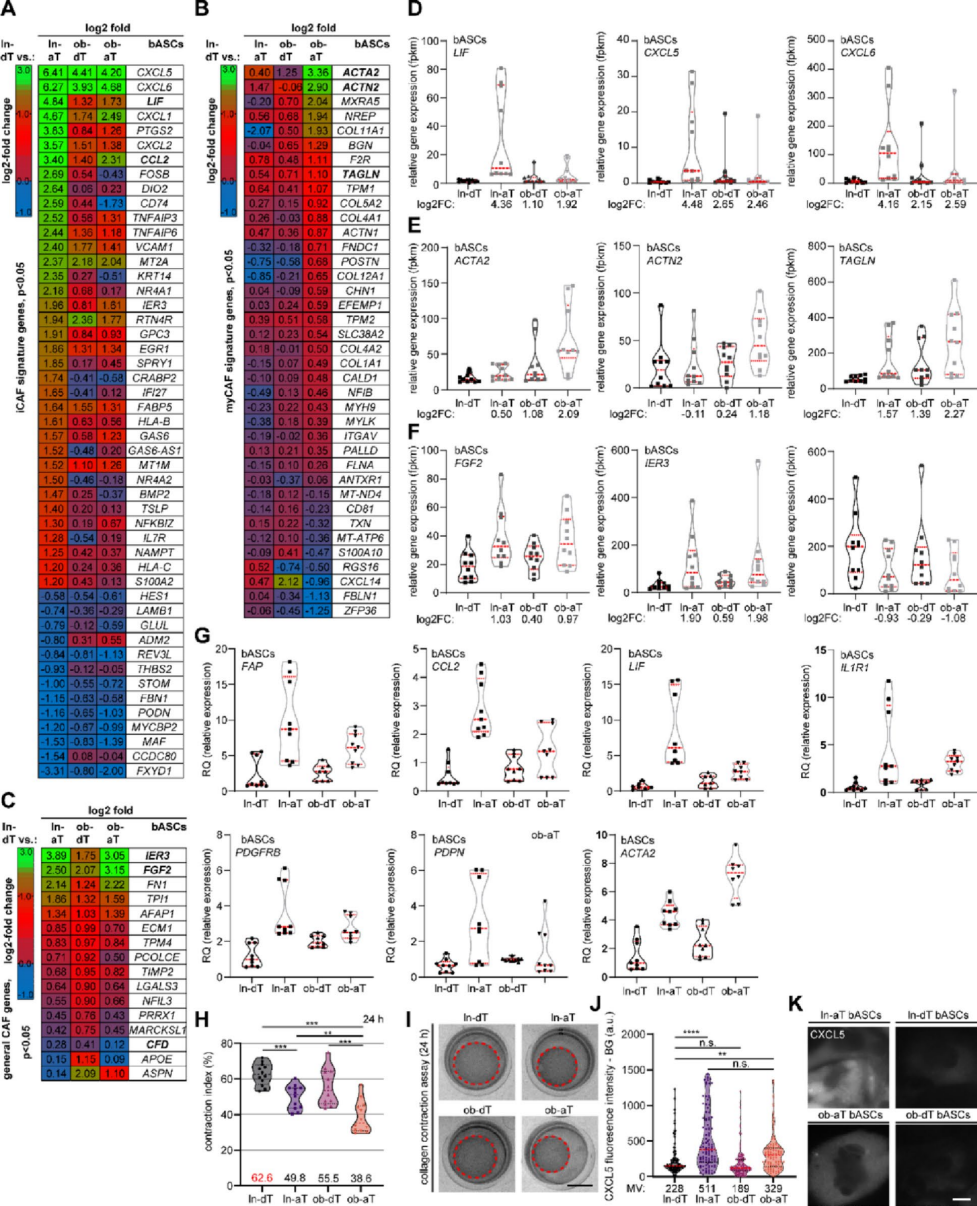
bASCs undergo de-differentiation into distinct CAF-like phenotypes with functional adaptations. **A**–**C** RNA-seq analysis of different bASCs subgroups (ln-dT, ln-aT, ob-dT, and ob-aT, five samples for each subgroup). Heatmaps depicting DEGs in iCAF-related (**A**), myCAF-related (**B**), and general CAF-related (**C**) genes in bASC subgroups. Comparisons include ln-dT vs. ln-aT, ln-dT vs. ob-dT, and ln-dT vs. ob-dT in all heatmaps. Log2-fold changes are represented by color intensity (green: + 3; red: + 1; blue: − 1). **D**–**F** Violin plots showing relative expression levels [fragments per kilobase per million mapped fragments (fpkm)] of selected CAF-subtype related DEGs. **D** iCAF-related DEGs, **E** myCAF-related DEGs, and **F** general CAF-related DEGs. The sample size of bASCs was ten for each subgroup (RNA-seq data were merged with a previously performed analysis [25]). **G** qPCR analysis of CAF-related genes depicted by violin plots, including *FAP*, *CCL2*, *LIF*, *IL1R1*, *PDGFRβ*, *PDPN*, and *ACTA2.* Results are displayed as RQ ± SEM from three independent experiments. **H** and **I** Functional assessment of gel contraction by bASC subgroups embedded in collagen matrices for 24 h. **H** Quantitative analysis of collagen gel contraction using the formula: contraction index = 100 * (circumference at time point/initial circumference). Results are presented as mean ± SEM (*n* = 6 biological samples, each measured in duplicates). **I** Representative images of contracted gels are shown. Scale bar, 5 mm. **J** and **K** Immunofluorescence staining of the iCAF marker CXCL5. **J** Violin plots showing quantification of cytoplasmic CXCL5 fluorescence intensity (arbitrary units, a.u.) in bASC subgroups. **K** Representative immunofluorescence images of CXCL5 staining. Scale bar, 20 μm. An unpaired Mann-Whitney U test was used in **H**. Student’s t-test was used in **G**. Statistical significance was assessed in **J** using the Kruskal-Wallis test followed by Dunn’s multiple comparisons test (adjusted *p* values reported). ∗*p* < 0.05, ∗∗*p* < 0.01, ∗∗∗*p* < 0.001, ∗∗∗∗*p* < 0.0001. No outlier test was performed for fpkm values to ensure better comparability between A-C and D-F

By actively remodeling their TME, tumors promote their progression, proliferation, metastasis, and resistance to therapy [16, 55]. CAFs, originating from various sources including fibroblasts and MSCs [56, 57], are integral components of the TME and play pivotal roles in its remodeling [58]. We have reported that breast cancer educates ln-aT bASCs to a higher extent into the iCAF-like phenotype, whereas ob-aT bASCs display an increased myCAF-like phenotype [25]. Based on a marker panel cited by recent studies [20, 29, 59, 60] and DrCAF (Data resource of Cancer-Associated Fibroblast) database [61], we identified a remarkable upregulation of 88 iCAF-related genes in ln-aT bASCs (Fig. 2A), notably leukemia inhibitory factor (*LIF*), *CXCL5*, and *CXCL6*, while 20 iCAF genes were significantly deregulated in ob-aT bASCs (Fig. 2A and D). On the other hand, ob-aT bASCs demonstrated a prominent expression of key myCAF markers, including actin alpha 2, smooth muscle (*ACTA2*), actinin alpha 2 (*ACTN2*), transgelin (*TAGLN*), and *COL11A1*, in contrast to a moderate upregulation of myCAF genes in lean counterparts (Fig. 2B and E). Additionally, more broad CAF genes associated with fibroblast activation and cellular reprogramming, such as immediate early response 3 (*IER3*), fibroblast growth factor 2 (*FGF2*), *FN1*, caveolin-1 downregulating factor (*CDF1*), were deregulated in both bASC subtypes (Fig. 2C and F).

To corroborate these findings, qPCR was performed with known CAF markers. The cells showed a significantly upregulated gene expression of multiple CAF markers, including *FAP*, and iCAF-related genes (*CCL2*, *LIF*, *IL1R1*, platelet derived growth factor receptor beta (*PDGFRβ)*, and podoplanin (*PDPN)*) in ln-aT bASCs relative to control ln-dT bASCs (Fig. 2G). Ob-aT bASCs similarly showed an increase in these markers, with a pronounced rise in the myCAF-associated gene *ACTA2* (Fig. 2G). To capture myCAF-specific activity, a collagen gel contraction assay [62] was performed. This assay revealed the collagen contraction capacity among four bASC subgroups with the following contraction indices of 61.2% in ln-dT, 48.4% in ln-aT, 53.7% in ob-dT, and 36.4% in ob-aT (Fig. 2H and I). Notably, the lowest free value and the resulting highest gel contraction capability were observed in ob-aT bASCs (Fig. 2H and I), highlighting a pronounced myCAF phenotype. Furthermore, immunofluorescence intensity analysis of the iCAF marker CXCL5 revealed a pronounced cytoplasmic upregulation in ln-aT bASCs (511 a.u.) relative to ln-dT bASCs (228 a.u.) (Fig. 2J and K). CXCL5 expression was also elevated in ob-aT bASCs (329 a.u.) compared with ln-dT bASCs, although to a substantially lesser extent than in ln-aT cells (Fig. 2J and K).

These data strengthen the hypothesis that the cancer-education process in the breast cancer TME is shaped by obesity-associated factors in a BMI-dependent manner (BMI < 25 vs. BMI ≥ 35), involving complex crosstalk between multiple stromal and immune cell types: while ln-aT bASCs predominantly adopt an iCAF-like profile characterized by upregulation of cytokine and chemokine genes, ob-aT bASCs undergo a shift toward a robust myCAF phenotype marked by elevated expression of *ACTA2*, *COL4A3*, and *COL11A1*, and enhanced gel contraction capacity.

### TGFβ drives myCAF-like de-differentiation in ob-dT bASCs, with obesity-associated upregulation of *TGFβ* gene expression across diverse cell types in the obese TME

**Fig. 3:**
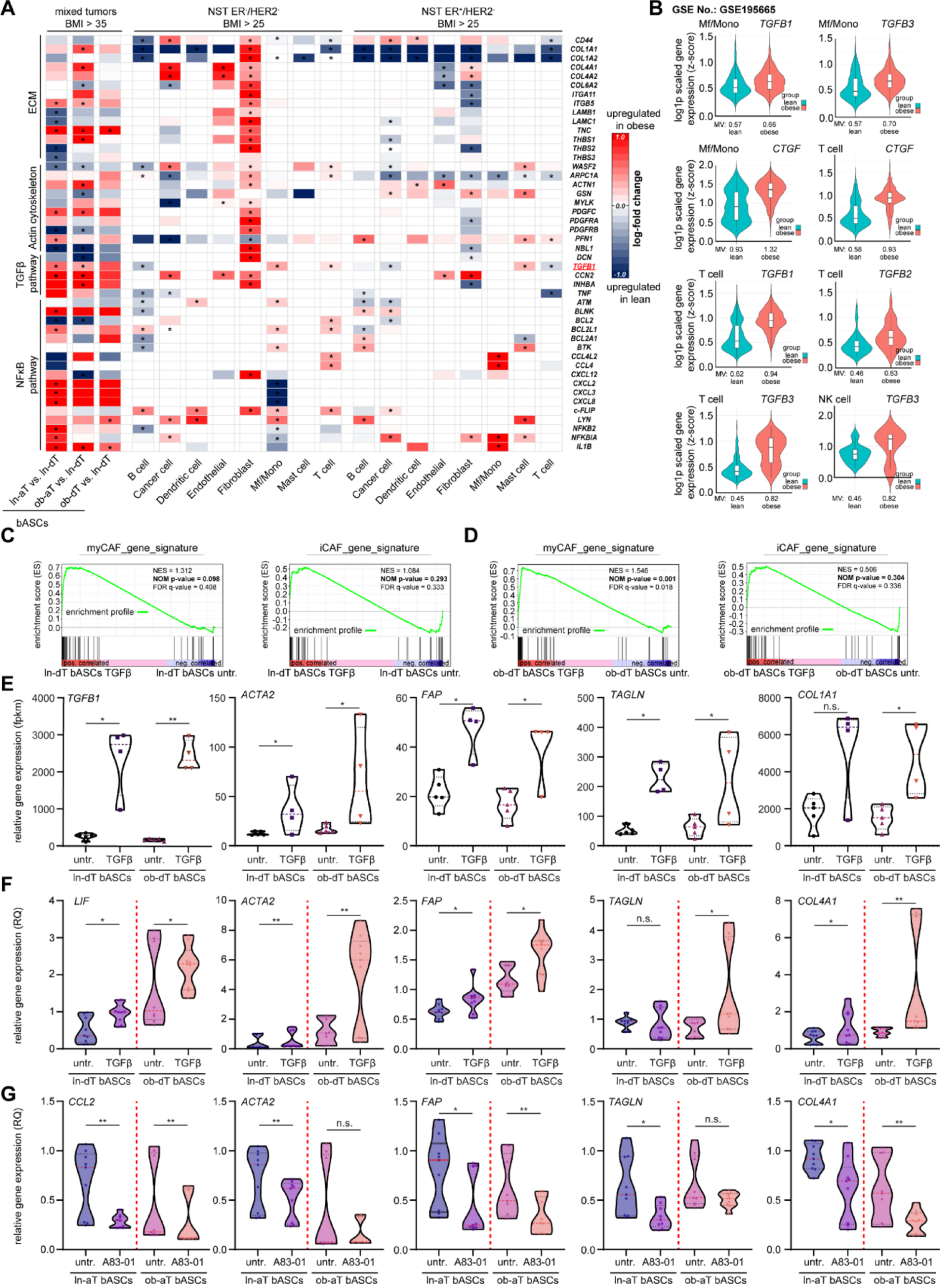
TGFβ drives ob-dT bASCs into a myCAF-like state and is elevated across immune and stromal compartments in the obese TME of breast cancer. **A** Single-cell RNA-seq data obtained from primary breast cancer tissues of the BioKey dataset from Bassez et al. [36] and BMI-dependent re-analyzed by Nguyen et al. [37] were used, focusing on breast cancer samples marked with NST ER^+^/HER2^−^ and NST ER^−^/HER2^−^. Heatmaps illustrating DEGs in bASCs subgroups (ln-aT vs. ln-dT, ob-aT vs. ln-dT, and ob-dT vs. ln-dT), and in eight cell types compared between patients with obesity (BMI > 30) and patients with normal weight (BMI < 25) in the NST ER^+^/HER2^−^ and NST ER^−^/HER2^−^ subgroups extracted from Nguyen et al. [37]. The cell color is scaled based on the log-FC (logarithmic fold change) values (obese vs. lean) estimated by the MAST test for NST ER^+^/HER2^−^ and NST ER^−^/HER2^−^ subgroups. Gray cells indicate their genes not being tested due to expression in less than 10% of the corresponding cell type in both BMI categories. *p* values shown were adjusted for multiple testing using the Benjamin-Hochberg method for NST ER^+^/HER2^−^ and NST ER^−^/HER2^−^ subgroups, presented as *q* values. *q < 0.05. Student’s t-test was used for bASC subgroups. ∗*p* < 0.05. **B** Violin plots showing BMI-stratified expression of key TGFβ pathway components and targets across indicated cell types in scRNA-seq data from human breast cancer patients, re-analyzed from the single-cell RNA-seq dataset GSE195665 [38]. Patients were grouped by BMI (obese: BMI ≥ 35; lean: BMI < 25), and gene expression levels are shown for *TGFB1*, *TGFB2*, *TGFB3*, and *CTGF* in macrophages/monocytes (Mf/Mono), CD4^+^ T cells, and NK cells. Expression is plotted as normalized log-transformed counts. Z-scores were computed from log-normalized expression values and represent relative gene expression across the dataset. Statistical significance was assessed using a two-sided Wilcoxon rank-sum test (adjusted *p* < 0.05). **C** and **D** GSEA was performed to compare myCAF and iCAF gene signatures in ln- (**C**) and ob-dT bASCs (**D**) treated with TGFβ (5 ng/ml for 3 days) vs. untreated controls. Enrichment was assessed using normalized enrichment scores (NES), nominal *p*-values, and false discovery rates (FDR), with significance defined as *p* < 0.05 and FDR < 0.25. **E**–**G** Violin plots depicting the relative expression levels of selected CAF subtype-related genes in ln- and ob-dT bASCs under the indicated treatment. **E** RNA-seq-based expression values (fpkm) following TGFβ treatment (5 ng/ml, 3 days) compared to untreated controls. **F** qPCR-based relative quantification (RQ) of DEGs under identical TGFβ treatment conditions. **G** qPCR-derived expression levels (RQ) of CAF-related DEGs in ln- and ob-dT bASCs treated with the TGFβ receptor inhibitor A83-01 (1 µM, 3 days) vs. controls. Statistical analyses were performed in R (version 2025.05.1) using the built-in statistics package. Two-tailed unpaired Mann-Whitney U tests are applied. *p < 0.05, **p < 0.01

To further investigate the relationship between obesity, TGFβ signaling, and stromal reprogramming, we examined a published scRNA-seq dataset from breast cancer patients by Bassez et al. [36], which Nguyen et al. [37] reanalyzed with respect to BMI categories according to the World Health Organization (WHO) criteria: normal weight (18.5 ≤ BMI < 25 kg/m²), overweight (25 ≤ BMI < 30 kg/m²), and obesity (BMI ≥ 30 kg/m²). This dataset includes no special type (NST) ER^+^/HER2^−^ breast cancer samples from six patients with normal weight, three with overweight, and four with obesity, as well as NST ER^−^/HER2^−^ breast cancer samples from eight patients with normal weight, two with overweight, and two with obesity [37]. Strikingly, comparing data from our isolated bASC subpopulations with their fibroblast clusters identified in the scRNA-seq dataset [37], we observed a highly similar transcriptional profile between ob-aT bASCs and fibroblasts derived from NST ER^−^/HER2^−^ breast cancers (Fig. 3AA, 2nd vs. 8th lane). Both populations exhibited an elevated expression of ECM components (*COL1A1*, *COL4A1*, *COL4A2*), integrins (*ITGA11*, *ITGB5*), and additional myCAF-associated markers, including *CCN2*, *INHBA*, and *IL1B*. In contrast, fibroblasts from NST ER^+^/HER2^−^ tumors lacked this transcriptional resemblance to ob-aT bASCs (Fig. 3AA, 2nd vs. 16th lane). These observations highlight that obesity induces comparable phenotypes shared by primary ob-aT bASCs as well as fibroblasts from NST ER^−^/HER2^−^ breast cancer samples.

Importantly, significantly elevated *TGFB1* expression was observed in macrophage/monocyte and T cell populations from NST ER^−^/HER2^−^ tumors, as well as in mast cells from ER^+^/HER2^−^ tumors (Fig. 3A and 9th, 11th, and 18th lanes), supporting a broad and cell-type-specific upregulation of TGFβ signaling within the obese TME. This finding is further substantiated by BMI-stratified analysis of another scRNA-seq breast cancer dataset (GSE195665) [38], which revealed a significantly increased expression of multiple key TGFβ pathway components, including *TGFB1*, *TGFB2*, *TGFB3*, and *CTGF* (connective tissue growth factor), in macrophages/monocytes (Mf/Mono), T cells, and NK cells from patients with obesity compared to lean individuals (Fig. 3B). These observations suggest an obesity-dependent activation of TGFβ signaling across multiple immune and stromal compartments, potentially contributing to the reprogramming of bASCs toward a myCAF-like phenotype.

To experimentally assess the sufficiency of TGFβ in driving this stromal remodeling, we stimulated ln- and ob-dT bASCs with recombinant TGFβ up to 3 days followed by bulk RNA-seq. GSEA revealed a non-significant increase in both myCAF and iCAF signatures in TGFβ-treated ln-dT bASCs (Fig. 3C). In contrast, TGFβ stimulation of ob-dT bASCs resulted in a robust and significant enrichment of the myCAF gene signature (NES: 1.546, NOM p-value: 0.001, FDR q-value: 0.018), with no concomitant induction of the iCAF program (Fig. 3D). Transcript-level analysis further confirmed an upregulation of specific myCAF-associated genes, including *TGFB1*, *ACTA2*, *FAP*, *TAGLN*, and *COL1A1* (Fig. 3E). This transcriptional activation was validated by qPCR, which showed consistence with a significant induction of *LIF*, *ACTA2*, *FAP*, *TAGLN*, and *COL4A1* in TGFβ-treated ob-dT bASCs (Fig. 3F).

To directly assess the dependency on TGFβ signaling, we inhibited ALK5 (activin receptor-like kinase 5), ALK4, and ALK7, serine/threonine kinase receptors mediating the TGFβ signaling pathway [63], using the small-molecule inhibitor A83-01 [64]. In both ln- and ob-dT bASC subtypes, A83-01 treatment resulted in a clear downregulation of key myCAF-associated genes, including *CCL2*, *ACTA2*, *FAP*, *TAGLN*, and *COL4A1* (Fig. 3G), underlining the requirement of canonical TGFβ signaling in the induction of the myCAF-like transcriptional state.

These findings collectively show that TGFβ is both sufficient and necessary to drive a myCAF-like reprogramming in ob-dT bASCs, whereas this effect does not occur in their lean counterparts within the same concentration and timeframe. In fact, scRNA-seq and bulk RNA-seq data suggest that elevated *TGFB1* expression in multiple immune and stromal cell types within the breast cancer TME likely contributes to this obesity-dependent stromal education. This mechanistic link between obesity and fibroblast lineage plasticity provides insight into how the metabolic state of the host may modulate tumor-stroma interactions.

### IL1RA or JAKi reprograms cancer-educated bASCs

Reprogramming the stromal cell compartment within the TME has emerged as a promising therapeutic strategy across multiple malignancies. In particular, reprogramming CAFs may help preserve or restore cancer-restraining CAF subpopulations [65], thereby limiting tumor progression. The key molecular pathways implicated in stromal reprogramming include IL1-, TGFβ-, and JAK1/2-signaling.

To assess the responsiveness of bASCs to the IL1α/β-JAK-STAT axis [27], control ln- and ob-dT bASCs were treated with either IL1α or LIF for up to 3 days, and quantitative PCR was performed to measure *LIF*, *CCL2*, *TAGLN*, and *COL4A1* expression. The downstream effectors of LIF, such as *BCL2*, *NANOG*, and *SOCS3* (suppressor of cytokine signaling 3), were also analyzed. Despite the proper induction of STAT3 (signal transducer and activator of transcription 3) signaling by LIF, as indicated by increased expression of *BCL2*, *NANOG*, and *SOCS3* in both ln- and ob-aT bASCs (Fig. S3A, 1st vs. 3rd and 5th vs. 7th violin plots), LIF stimulation did not elicit significant changes in the expression of *LIF*, *CCL2*, *TAGLN*, or *COL4A1* (Fig. S3B, 1st vs. 3rd and 4th vs. 6th violin plots). In contrast, IL1α treatment resulted in a marked upregulation of *LIF* and *CCL2*, consistent with activation of the IL1 signaling axis. Notably, expression of the myCAF-associated genes *TAGLN* and *COL4A1* was modestly reduced upon IL1α stimulation (Fig. S3B, 1st vs. 2nd and 4th vs. 5th violin plots), suggesting a potential shift away from a myCAF-like phenotype under pro-inflammatory stimuli.

To determine whether cancer-educated bASCs could be reprogrammed toward a cancer-suppressive phenotype, ln- and ob-aT bASCs were exposed to IL1RA anakinra, JAKi AZD1480, or TGFβ for up to 3 days. Total RNAs were extracted for RNA-seq. Common gene and pathway alterations were examined in both ln- and ob-aT bASCs. IL1RA treatment resulted in a significant downregulation of key inflammatory signaling genes in both bASC subtypes, particularly in ln-aT cells, such as RELB proto-oncogene, NF-ĸB subunit (*RELB)*, *LIF*, *CXCL1*, and *CXCL8* (Fig. S3C and G). This was accompanied by the suppression of tumor necrosis factor (TNF), IL-17, and NF-κB signaling pathways (Fig. S3E). Intriguingly, JAKi induced more extensive transcriptional changes in both ln- and ob-aT bASCs, markedly downregulating JAK-STAT, interferon (IFN), and MAPK signaling pathways (Fig. S3D and F). This downregulation involved key downstream effectors of JAK1/2 signaling, such as *SOCS3*, *STAT3*, interferon regulatory factor 7 (*IRF7)*, and interferon induced transmembrane protein 3 (*IFITM3)* (Fig. S3D, F, H). These findings underscore the pathway-specific impact of these inhibitors on bASCs, emphasizing their potential for targeted modulation of stromal cells within the TME.

**Fig. 4:**
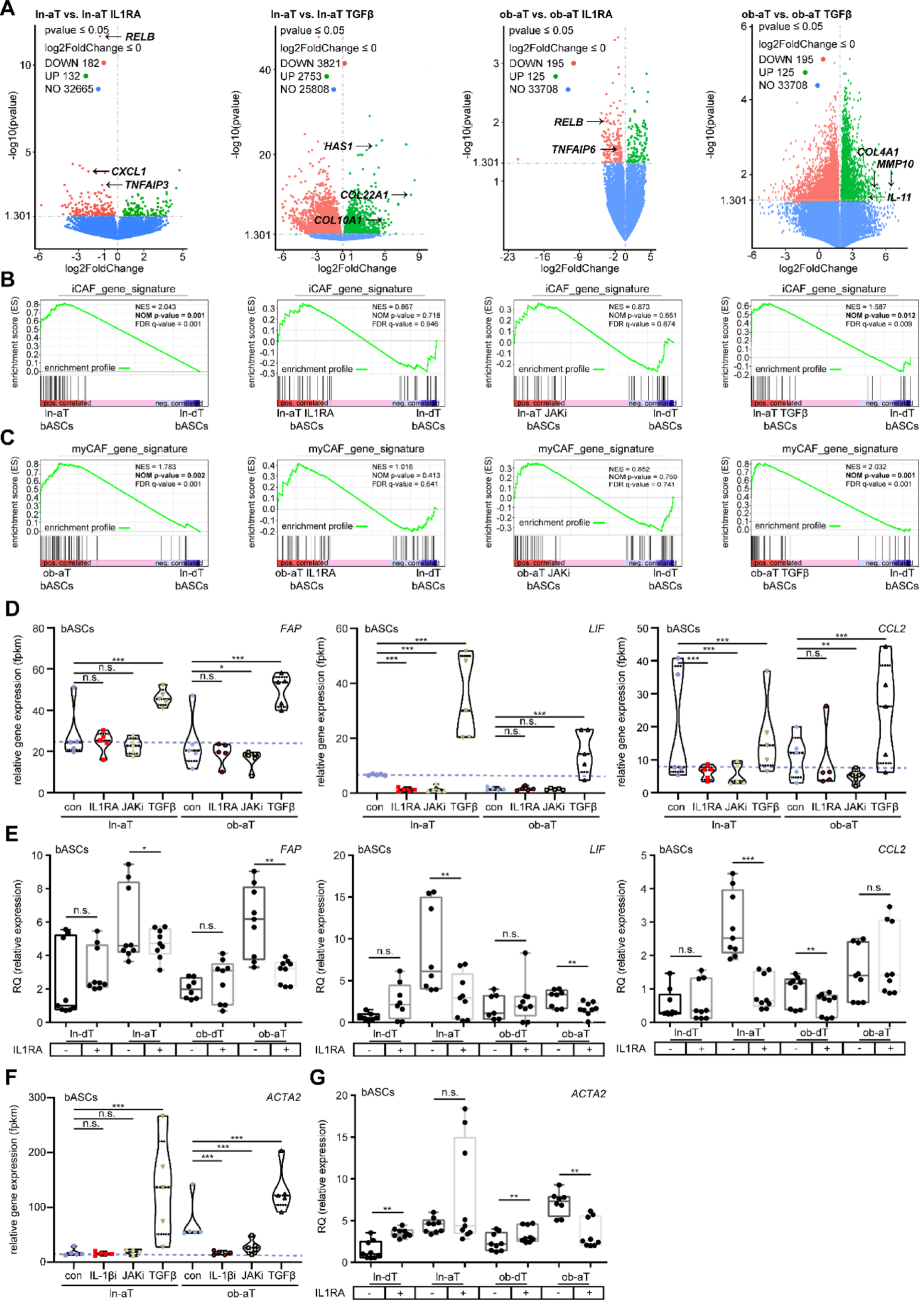
IL1RA is sufficient to reprogram iCAF- and myCAF-like phenotypes, whereas TGFβ stimulates both phenotypes. **A**, **D**, and **F** RNA-seq analysis of ln- and ob-aT bASC subgroups treated with DMSO, IL1RA anakinra, JAKi AZD1480, or TGFβ. **A** Volcano plots show differentially expressed genes (DEGs) between specific subgroups: ln-aT vs. ln-aT bASCs treated with IL1RA (1st panel), ln-aT vs. ln-aT bASCs treated with TGFβ (2nd panel), ob-aT vs. ob-aT bASCs treated with IL1RA (3rd panel), and ob-aT vs. ob-aT bASCs treated with TGFβ (4th panel). DEGs are plotted with adjusted *p*-values (y-axis) and log2 fold changes (x-axis). Upregulated genes are highlighted in green, downregulated genes in red, and non-significant genes in blue. Analyses were performed using the DESeq2 R package. **B** and **C** Gene set enrichment analysis (GSEA) plots displaying the normalized enrichment score (NES), nominal *p*-value (NOM *p*-value), and false discovery rate *q*-value (FDR *q*-value). **B** Illustrates the enrichment of an iCAF gene signature in ln-aT bASCs (ln‐aT, IL1RA, JAKi, and TGFβ) compared to ln‐dT bASCs. **C** Presents the corresponding analysis for a myCAF gene signature in ob‐aT bASCs (ob‐aT, IL1RA, JAKi, and TGFβ) vs. ln‐dT bASCs. **D** and **F** Violin plots representing RNA-seq-derived relative expression levels (fpkm) of CAF-related genes in treated bASC subgroups. **D** Expression levels of *FAP*, *LIF*, and *CCL2* in ln- and ob-aT bASCs treated with IL1RA, JAKi, or TGFβ. **F** Expression of *ACTA2* in bASC subgroups. **E** and **G** Quantitative PCR validation of RNA-seq data for CAF-related genes. Violin plots display relative expression levels of *FAP*, *LIF*, and *CCL2* in control bASCs and IL1RA-treated bASCs (**E**). Analysis of the myCAF marker ACTA2 is shown for IL1RA-treated bASCs (**G**). Student’s t-test was used in (**E**) and (**G**). ∗*p* < 0.05, ∗∗*p* < 0.01, ∗∗∗*p* < 0.001

We next assessed whether inhibitor treatment could reverse the cancer-educated phenotype of bASCs. ln-aT bASCs treated with IL1RA or JAKi demonstrated a substantial reduction in the expression of multiple iCAF genes, including *CXCL1*, *CXCL3*, *CXCL5*, *CXCL6*, *CXCL10*, *IL1B*, *RELB*, and *LIF* (Fig. 4A; Figs. S3G; S4A and C). Intriguingly, IL1RA and JAKi also interfered with the myCAF phenotype in ob-aT bASCs, with a substantial downregulation of myCAF-associated markers, such as *HAS1*, *COL4A1*, tenascin C (*TNC)*, serpin family E member 1 (*SERPINE1)*, and *CNN1* (Fig. S4B and D). As a control, TGFβ stimulation induced a robust increase in cytokine secretion profile in ln-aT bASCs, including *IL6*, *IL11*, *CCL2*, and additional inflammatory mediators (Fig. S4A, 4th lane), as well as myCAF-associated gene expression (Fig. S4B and D, 4th lane and 5th violin plot), reinforcing its fundamental role in initiating and maintaining CAF-like phenotypes. Furthermore, by leveraging GSEA with curated iCAF and myCAF gene signatures (Supplementary Material 2), we corroborated the robust induction of the iCAF phenotype in ln-aT bASCs (normalized enrichment score (NES) = 2.043, NOM *p* = 0.001, FDR q = 0.001; Fig. 4B, 1st profile). Notably, treatment with either IL1RA or a JAK inhibitor effectively reprogrammed this de-differentiation process (IL1RA: NES = 0.887, NOM p-value = 0.718, FDR q-value = 0.946; JAKi: NES = 0.873, NOM p-value = 0.661, FDR q-value = 0.874), whereas TGFβ, despite altering the overall transcriptomic profile, failed to suppress the iCAF signature (Fig. 4B, 2nd – 4th profiles). In parallel, untreated ob‐aT bASCs exhibited a pronounced myCAF signature (NES = 1.783, NOM p-value = 0.002, FDR q-value = 0.001), which was completely abrogated by either of the inhibitors (Fig. 4C, profiles 1st–3rd profiles). As anticipated, TGFβ displayed the same tendency as in ob-dT bASCs (Fig. 3B–F), further amplifying the myCAF gene signature (NES = 2.032, NOM p-value = 0.001, FDR q-value = 0.001; Fig. 4C, 4th profile). These findings demonstrate that both cancer-educated phenotypes, iCAF and myCAF, can be effectively reprogrammed by targeting IL1β or JAK signaling pathways.

**Fig. 5:**
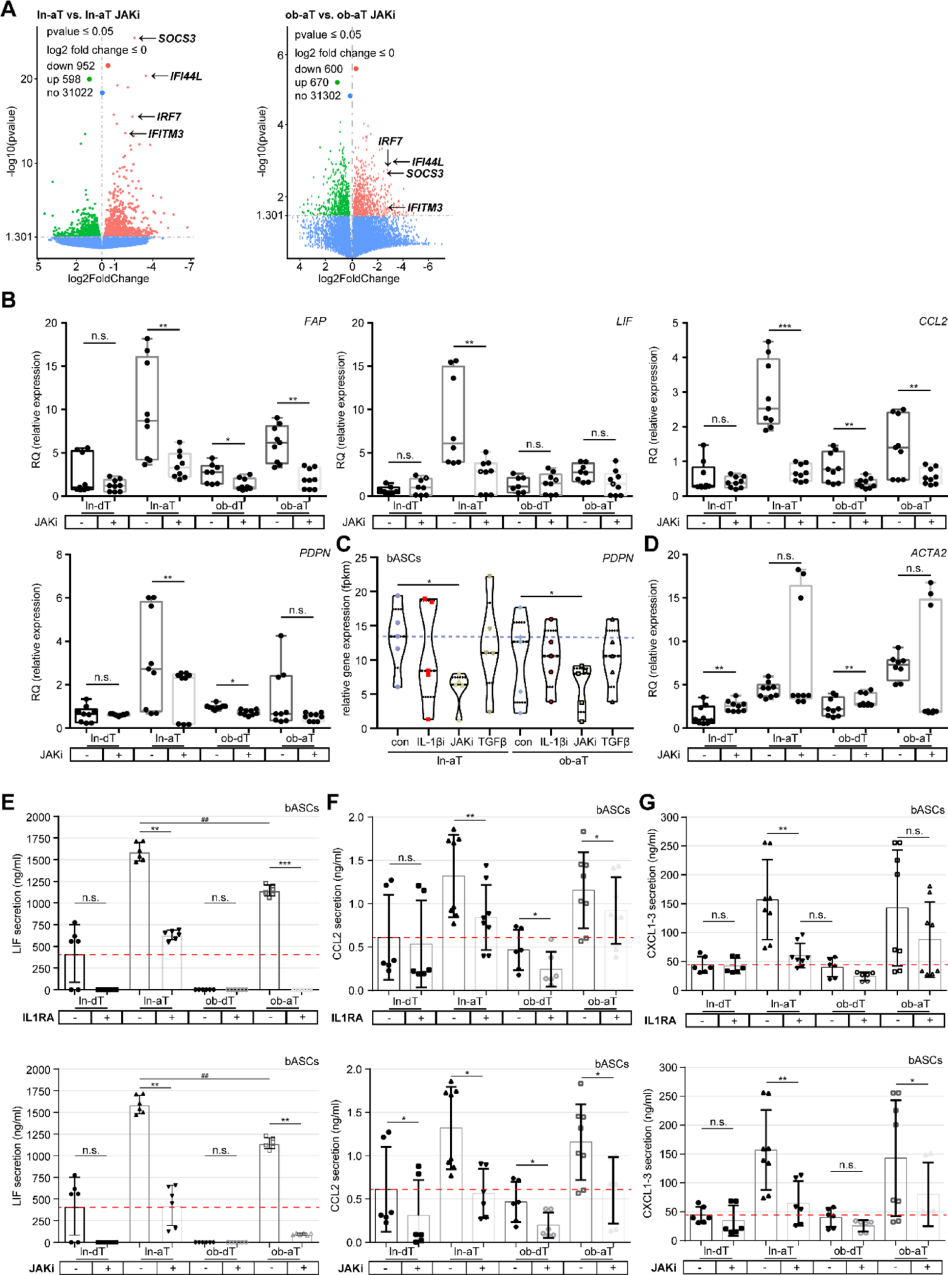
Either IL1RA or JAKi is sufficient to reprogram iCAF- and myCAF-like phenotypes. **A** and **C** RNA-seq analysis of ln- and ob-aT bASC subgroups treated with DMSO or inhibitors (anakinra, AZD1480, TGFβ) as indicated. **A** Volcano plots show differentially expressed genes (DEGs) between subgroups: ln-aT vs. ln-aT bASCs treated with JAKi (1st panel), and ob-aT vs. ob-aT bASCs treated with JAKi (2nd panel). DEGs are plotted with adjusted *p*-values (y-axis) and log2 fold changes (x-axis). Upregulated genes are highlighted in green, downregulated genes in red, and non-significant genes in blue. Analyses were performed using the DESeq2 R package. **C** Violin plot representing relative gene levels (fpkm) of *PDPN* from RNA-seq analysis of treated bASC subgroups. **B** and **D** CAF-related gene levels via qPCR. Violin plots display relative gene levels of (**B**) *FAP*, *LIF*, *CCL2*, and *PDPN* in bASC control and JAKi-treated bASCs. Analysis of the myCAF marker (D) ACTA2 is shown for JAKi-treated bASCs. **EG** ELISA analysis of cytokine and chemokine levels in conditioned media from bASC subgroups treated with the indicated inhibitor for 72h. Quantification of *LIF* (**E**), *CCL2* (**F**), and *CXCL1-3* (**G**) was performed for control bASCs compared to those treated with IL1RA (upper panels) or JAKi (lower panels). The results are from three independent experiments and presented as scatter bar graphs with meanSEM. Students t-test was used in (**B**, **DG**). *p*0.05, *p*0.01, *p*0.001

To underline these findings, we further analyzed phenotype-related gene expression levels of *FAP*, *CCL2*, *LIF*, *PDPN*, *IL1R1*, and *PDGFRβ* using both RNA-seq and qPCR. The data revealed a considerable downregulation of *CCL2*, *PDPN*, *IL1R1*, and *LIF* in ln-aT bASCs following the IL1RA treatment in both gene analyses (Fig. 4A and D; Fig. S5C, E, F), while the reduction in *FAP* expression was more pronounced in the qPCR gene analysis (Fig. 4E, 1st scatter boxplot). In ob-aT bASCs, a modest reduction in *CCL2*, *PDPN*, and *LIF* was observed, with a more substantial decrease in *FAP* expression (Fig. 4D and E, 1st plots; Fig. S5C). Additionally, TGFβ treatment significantly increased gene levels of *FAP*, *LIF*, and *ACTA2* (Fig. 4F and Fig. S5A and B), but not those of *PDPN*, *IL1R1*, or *PDGFRβ* (Fig. S5D, E, G, I and K). Consistent with these observations, *ACTA2* was notably downregulated in ob-aT bASCs upon IL1RA treatment (Fig. 4F and G). JAKi treatment strongly downregulated iCAF-related genes, including *LIF*, *CCL2*, *PDPN*, and *IL1R1* (Fig. 5B and C; Fig. S5E and H), with effects on gene expression even more pronounced than those of IL1RA (Fig. 4E vs. Fig. 5B). Interestingly, while JAKi treatment also led to a significant reduction in *FAP* expression, it did not affect *ACTA2* levels in the qPCR gene analysis data (Fig. 5B and D). *PDGFRβ*, used as a marker in a CAF study from colorectal cancer patients [29], showed inconclusive results (Fig. S5I-L).

Finally, to examine the functional reprogramming of bASC subtypes, ELISA assays were conducted on IL1RA- and JAKi-treated bASCs. Defined cell numbers were seeded, and supernatants were collected after 72 h of incubation. The results were consistent with RNA-seq and qPCR findings, revealing a significant reduction in cytokine secretion of LIF (Fig. 5E), CCL2 (Fig. 5F), and CXCL1-3 (Fig. 5G) in response to IL1RA (upper panels) or JAKi treatment (lower panels) in both ln- and ob-aT bASC subtypes.

Our results show that targeting IL1 and JAK signaling effectively reprograms cancer-educated bASC phenotypes, probably reducing their cancer-supportive functions within the TME. The treatment with IL1RA or JAKi downregulated key genes related to iCAF and myCAF phenotypes and significantly decreased cytokine secretion. In contrast, TGFβ promoted the cancer-supportive phenotype, enhancing iCAF- and myCAF-related gene expression as presented for ln- and ob-dT bASCs.

### Reprogrammed ln- and ob-aT bASCs markedly reduce their capacity to induce EMT in epithelial breast cancer cells

Given that MSCs primarily induce EMT in epithelial breast cancer cells via the secretion of bioactive factors [16, 32], we hypothesized that reprogramming the iCAF population would diminish the induction of EMT in these cells. To examine this issue, ln- and ob-aT bASCs, treated with DMSO, IL1RA, or JAKi for 72 h, were co-cultured with epithelial breast cancer cells MCF7 for up to 14 days, as reported [25]. Following co-culture, the cells were sorted using epithelial marker CD24 and mesenchymal marker CD73 (Fig. S6A) for further analyses.

**Fig. 6:**
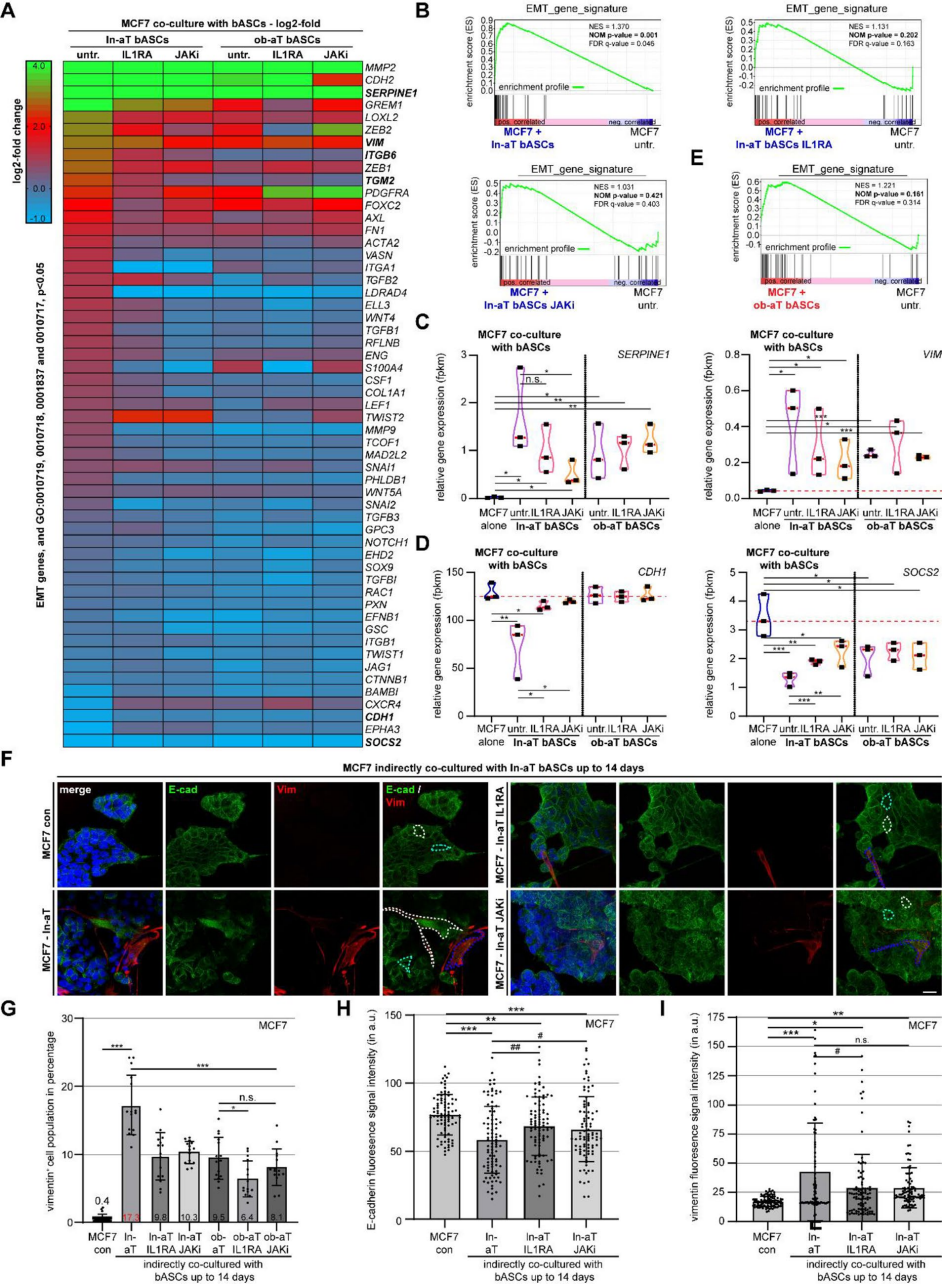
Reprogrammed ln- and ob-aT bASCs demonstrate a significantly compromised ability to drive EMT in MCF7 cells. **A**–**E** RNA-seq analysis of MCF7 cells cultured alone or in direct co-culture with different bASC subgroups with or without pretreatment as indicated (ln-aT, ln-aT IL1RA, ln-aT JAKi, ob-aT, ob-aT IL1RA, and ob-aT JAKi) for 14 days (*n* = 3). Following co-culture, cells were FACS-sorted using epithelial marker CD24 and mesenchymal marker CD73. The gene expression was analyzed using DESeq2 R package. **A** Heatmap of DEGs in EMT pathways (GO: 0010719, 0001837, 0010717, 0010718) in co-cultured MCF7 cells. Comparisons include MCF7 alone vs. ln-aT bASCs, ln-aT bASCs treated with IL1RA, ln-aT bASCs treated with JAKi (1st–3rd panels) and MCF7 alone vs. ob-aT bASCs, ob-aT bASCs treated with IL1RA, and ob-aT bASCs treated with JAKi (4th -6th panels). Log2-fold changes are represented by color intensity (green: +4; red: +2; blue: − 1). **B** and **E** Gene set enrichment analysis (GSEA) plots displaying the normalized enrichment score (NES), nominal *p*-value (NOM *p*-value), and false discovery rate *q*-value (FDR *q*-value). Panels illustrate the enrichment of an EMT gene signature in MCF7 co-cultured with ln-aT bASCs subgroups (ln‐aT, IL1RA, JAKi) (**B**) or ob-aT bASCs compared to MCF7 untreated (**E**). **C** and **D** Violin plots displaying RNA-seq-derived relative gene expression levels (fpkm) of key mesenchymal genes (*SERPINE1*, *VIM*) (**C**) and epithelial gene/EMT negative regulator (*CDH1*, *SOCS2*) (**D**) in MCF7 cells co-cultured with indicated bASC subgroups. **F**–**I** MCF7 cells cultured alone or in indirect co-culture with bASCs with indicated treatment (ln-aT, ln-aT IL1RA, ln-aT JAKi) for 14 days were stained for E-cadherin (E-cad, epithelial marker, in green), vimentin (VIM, mesenchymal marker, in red), and DNA (DAPI, blue). **F** Representative images of MCF7 cells cultured alone or with indicated bASC subtypes. Scale bar, 25 μm. White dotted lines indicate areas used for cell size measurements, blue dotted lines depict areas for vimentin intensity measurements, and cyan lines mark regions for E-cadherin intensity measurements. **G**–**I** Vimentin positive cell population (**G**), E-cadherin fluorescence intensity (**H**), vimentin fluorescence intensity (**I**) of MCF7 cells after co-culture with bASCs. Data represent three independent experiments (*n* = 3, 90 cells per group) and are presented as scatter bar plots showing mean ± SEM. Statistical significance was determined using an unpaired Mann-Whitney U test. *p < 0.05, **p < 0.01, ***p < 0.001

RNA-seq analysis identified hundreds of differentially expressed genes in MCF7 cells co-cultured with non-treated or treated bASCs (ln-aT bASCs, ln-aT bASCs IL1RA, and ln-aT bASCs JAKi) compared to control MCF7 cells (Fig. 6A and S6B). Notably, MCF7 cells co-cultured with non-treated ln-aT bASCs displayed a significant EMT signature (Fig. 6B, NES = 1.370, NOM p-value = 0.001, FDR q-value = 0.046), with an upregulated expression of mesenchymal genes, such as *SERPINE1*, vimentin (*VIM)*, transglutaminase 2 (*TGM2)*, *ITGB6*, and matrix metalloproteinase-2 (*MMP2)* (Fig. 6A and C; Fig. S6B and D) and downregulated epithelial marker cadherin 1 (*CDH1)* and EMT negative regulator *SOCS2* (Fig. 6D; Fig. S6B). Co-culture of bASCs treated with IL1RA or JAKi effectively reduced EMT in MCF7 cells, as evidenced by a decreased EMT gene signature, decreased expression of mesenchymal genes, and normalized epithelial gene expression (Fig. 6A–D). Furthermore, pathway analysis revealed a significant decrease in genes associated with EMT regulation following inhibitor treatment (Fig. S6C). Consistently, both inhibitor-treated ln-bASC subgroups exhibited a marked reduction in EMT gene signature enrichment (Fig. 6B). As previously reported [25], ob-aT bASCs exhibited a limited capability to induce EMT, displayed by a non-significant EMT signature gene enrichment (Fig. 6E, NES = 1.221, NOM p-value = 0.161, FDR q-value = 0.314) and only a moderate increase in *SERPINE1*, *VIM*, and *TGM2* expression, but no significant changes in *ITGB6*, *CDH1*, or *SOCS2* expression (Fig. 6A and C–E, and Fig. S6D). Intriguingly, ob-aT bASCs treated with either IL1RA or JAKi were not able to significantly alter the gene expression profile in MCF7 cells (Fig. 6A, and C–E).

To assess EMT at the protein level, control MCF7 cells or MCF7 cells co-cultured with bASCs subgroups were stained for the mesenchymal marker vimentin and the epithelial marker E-cadherin. Quantification of vimentin-positive cells supported the data from the RNA-seq, showing a marked EMT induction in MCF7 cells with 17.3% vimentin-positive cells, compared to 0.4% in control MCF7 cells (Fig. 6F and G). The positive population was reduced to 9.8% and 10.3% upon treatment of ln-aT bASCs treated with IL1RA and JAKi, respectively (Fig. 6F and G). Ob-aT bASCs induced EMT in only 9.5% of MCF7 cells, whereas IL1RA further reduced this to 6.4% and JAKi had no additional effect (Fig. 6G and S6E). Moreover, ln-aT bASCs demonstrated a reduced fluorescence intensity of E-cadherin, an increased intensity of vimentin, and an enlarged cell size (Fig. 6F–I; Fig. S6I), which were significantly reversed by the IL1RA or JAKi treatment (Fig. 6F–I; Fig. S6I). In contrast, ob-aT bASCs treated with IL1RA or JAKi reduced the cell size (Fig. S6E and H), and treatment with IL1RA normalized the E-cadherin fluorescence intensity (Fig. S6F). Neither inhibitor was able to affect vimentin fluorescence intensity in MCF7 cells co-cultured with treated ob-aT bASCs (Fig. S6E and G). These results underscore the potential of IL1 and JAK pathway inhibitors to modulate EMT induction in epithelial breast cancers, particularly in iCAF-enriched stromal populations within the TME.

### Reprogramming of myCAF-like ob-aT bASCs suppresses CSC formation via Attenuation of extrinsic CSC-promoting signals

We demonstrated that ob-aT bASCs were capable of inducing CSC traits in the TNBC cell line MDA-MB-231 [25]. However, the specific molecular mechanisms, particularly the contribution of myCAF-like bASCs, remained poorly defined. To dissect the underlying signaling pathways and assess whether pharmacologic blockade of IL1 or JAK1/2 signaling could reverse the CSC-promoting effect, we conducted transcriptomic profiling.

**Fig. 7:**
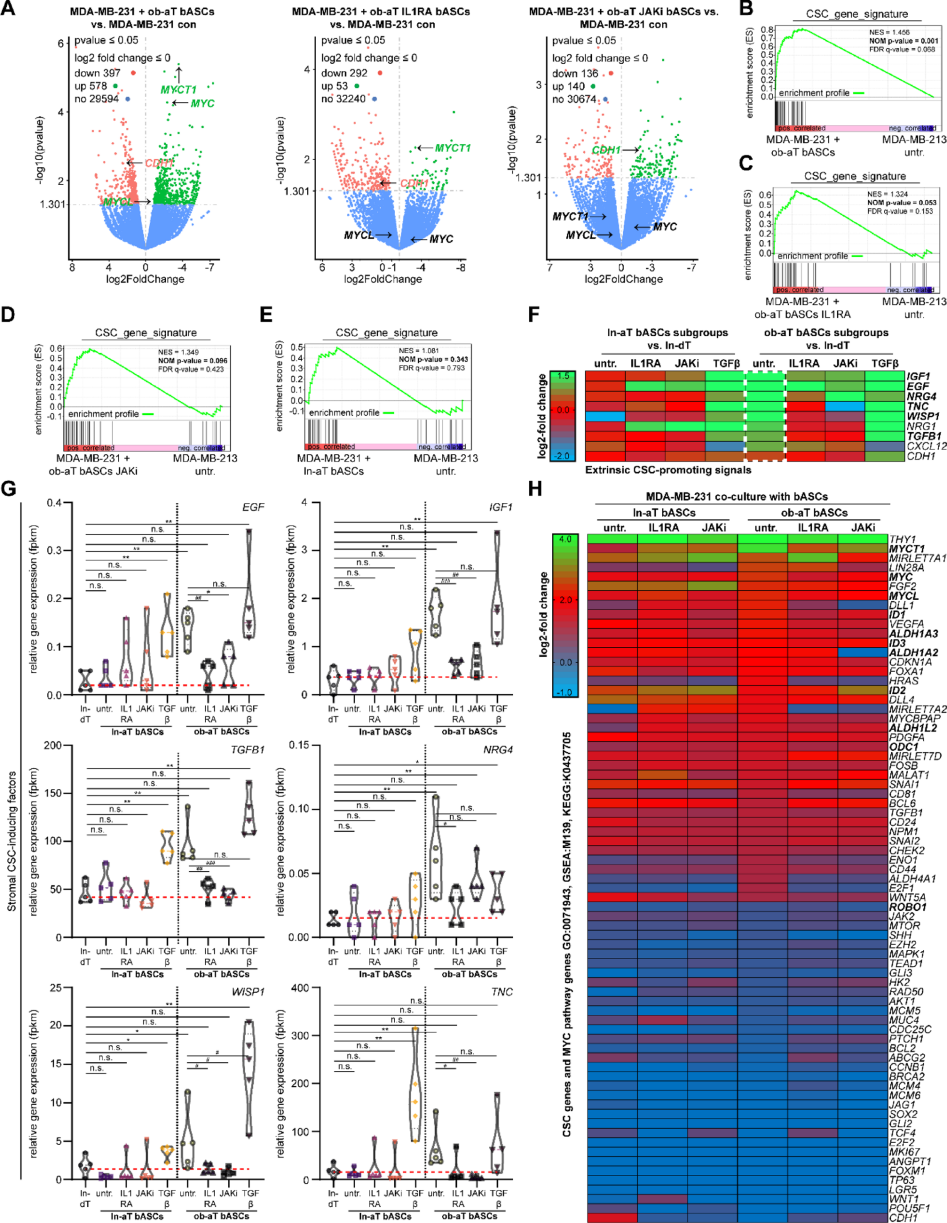
IL1RA or JAKi reprograms ob-aT bASCs and suppresses their ability to induce cancer stem cell traits in triple-negative breast cancer cells. **A**–**E** RNA-seq analysis of MDA-MB-231 cells, triple negative breast cancer cells, cultured alone or in direct co-culture with bASC subgroups treated as indicated (ln-aT, ln-aT IL1RA, ln-aT JAKi, ob-aT, ob-aT IL1RA, ob-aT JAKi) for 14 days (*n* = 3). Cells were subsequently sorted using the mesenchymal marker CD90. Differential gene expressions were analyzed using the DESeq2 R package. **A** Volcano plots show DEGs between specific subgroups: MDA-MB-231 + ob-aT bASCs vs. MDA-MB-231 alone (1st ), MDA-MB-231 + ob-aT bASCs treated with IL1RA vs. MDA-MB-231 alone (2nd ), and MDA-MB-231 + ob-aT bASCs treated with JAKi vs. MDA-MB-231 alone (3rd ). DEGs are visualized with adjusted *p*-values (y-axis) and log2 fold changes (x-axis). Upregulated genes are marked in red, downregulated in green, and non-significant genes in blue. **B**–**E** Gene set enrichment analysis (GSEA) plots showing the enrichment score of a defined CSC signature in MDA-MB-231 cells co-cultured with untreated ob-aT bASCs (**B**), IL1RA-treated ob-aT bASCs (**C**), JAKi-treated ob-aT bASCs (**D**), or untreated ln-aT bASCs (**E**). Plots display the normalized enrichment score (NES), nominal *p*-value (NOM *p*-value), and false discovery rate *q*-value (FDR *q*-value) and their enrichment profile. **F** Heatmap of DEGs associated with extrinsic CSC-promoting signals in the following bASCs subgroups: ln-aT untreated, ln-aT IL1RA, ln-aT JAKi, ln-aT TGFβ, ob-aT untreated, ob-aT IL1RA, ob-aT JAKi, and ob-aT TGFβ. **G** Violin plots displaying RNA-seq-derived relative expression levels (fpkm) of genes associated with extrinsic CSC-promoting signals (*EGF*, *IGF1*, *TGFB1*, *NRG4*, *WISP1*, *TNC*) in indicated bASCs subgroups. **H** Heatmap of DEGs in CSC formation and MYC pathways (GO: 0071943, GSEA: M139, KEGG: K0437705) in MDA-MB-231 cells alone or co-cultured as indicated. Log2-fold changes are represented by color intensity (green: +4; red: +2; blue: -1). Genes are considered differentially expressed if the adjusted *p*-value < 0.05 and log2 (fold change) > 0.5. ∗*p* < 0.05, ∗∗*p* < 0.01, ∗∗∗*p* < 0.001

Ob-aT and ln-aT bASCs were pretreated with either IL1RA or JAKi for 72 h prior to co-culture with MDA-MB-231 cells for up to 14 days. Both treated bASCs and tumor cells were subsequently separated based on differential expression of the mesenchymal marker CD90, which is robustly expressed in bASCs (fragments per kilobase per million mapped fragments (fpkm): ln-dT, 81.5) and nearly absent in MDA-MB-231 cells (fpkm: 0.015). Transcriptomic analysis of sorted MDA-MB-231 cells revealed 975 DEGs upon co-culture with untreated ob-aT bASCs, compared to 345 and 276 DEGs when co-cultured with IL1RA- or JAKi-pretreated ob-aT bASCs, respectively (Fig. 7A), suggesting an evident dampening of transcriptional reprogramming in TNBC cells following the pathway inhibition.

To assess the functional significance of this altered gene expression, we applied a curated CSC gene signature (Supplementary Material 2). GSEA revealed a strong enrichment of CSC-related transcripts in MDA-MB-231 cells co-cultured with untreated ob-aT bASCs (NES = 1.456, *p* = 0.001, FDR q = 0.068), whereas such alterations were largely absent in cancer cells co-cultured with ln-aT bASCs (NES = 1.081, *p* = 0.343, q = 0.793) (Fig. 7B and E). Crucially, pretreatment of ob-aT bASCs with IL1RA or JAKi reduced CSC signature enrichment to non-significant levels (IL1RA: NES = 1.324, *p* = 0.053, q = 0.153; JAKi: NES = 1.349, *p* = 0.096, q = 0.423) (Fig. 7C and D), demonstrating that inhibition of inflammatory signaling abrogates the CSC-inducing capacity of ob-aT bASCs.

Examination of the bASC transcriptomes revealed that a panel of known CSC-promoting factor genes, *IGF1* (insulin-like growth factor), *EGF* (epidermal growth factor), *TGFB1*, *NRG4* (neuregulin 4), *WISP1* (WNT1 inducible signaling pathway protein 1), and *TNC*, were highly upregulated in ob-aT bASCs relative to ln-aT bASCs and ln-dT bASCs (Fig. 7F and G). IL1RA or JAKi treatment dramatically downregulated these genes in ob-aT bASCs (Fig. 7F and G). Remarkably, ln-aT bASCs expressed these genes at minimal levels, whereas treatment with TGFβ induced their expression in both ln- and ob-aT bASCs (Fig. 7G), suggesting that TGFβ signaling can amplify the CSC-supportive niche. Given that many of these factors activate MYC signaling [66], we next examined MYC pathway activation in the breast cancer cells. Heatmap analysis revealed robust induction of CSC- and MYC-associated gene sets (GO:0071943; GSEA: M139; KEGG: K0437705) in MDA-MB-231 cells co-cultured with untreated ob-aT bASCs, whereas co-culture with inhibitor-pretreated bASCs led to downregulation of these pathways (Fig. 7H). These data indicate that ob-aT bASCs promote CSC formation at least in part through extrinsic activation of MYC signaling, and that pharmacologic reprogramming of ob-aT bASCs suppresses this CSC-inductive niche.

### Blockade of IL1R or JAK1/2 signaling suppresses MYC-driven CSC programs and ALDH activity

**Fig. 8:**
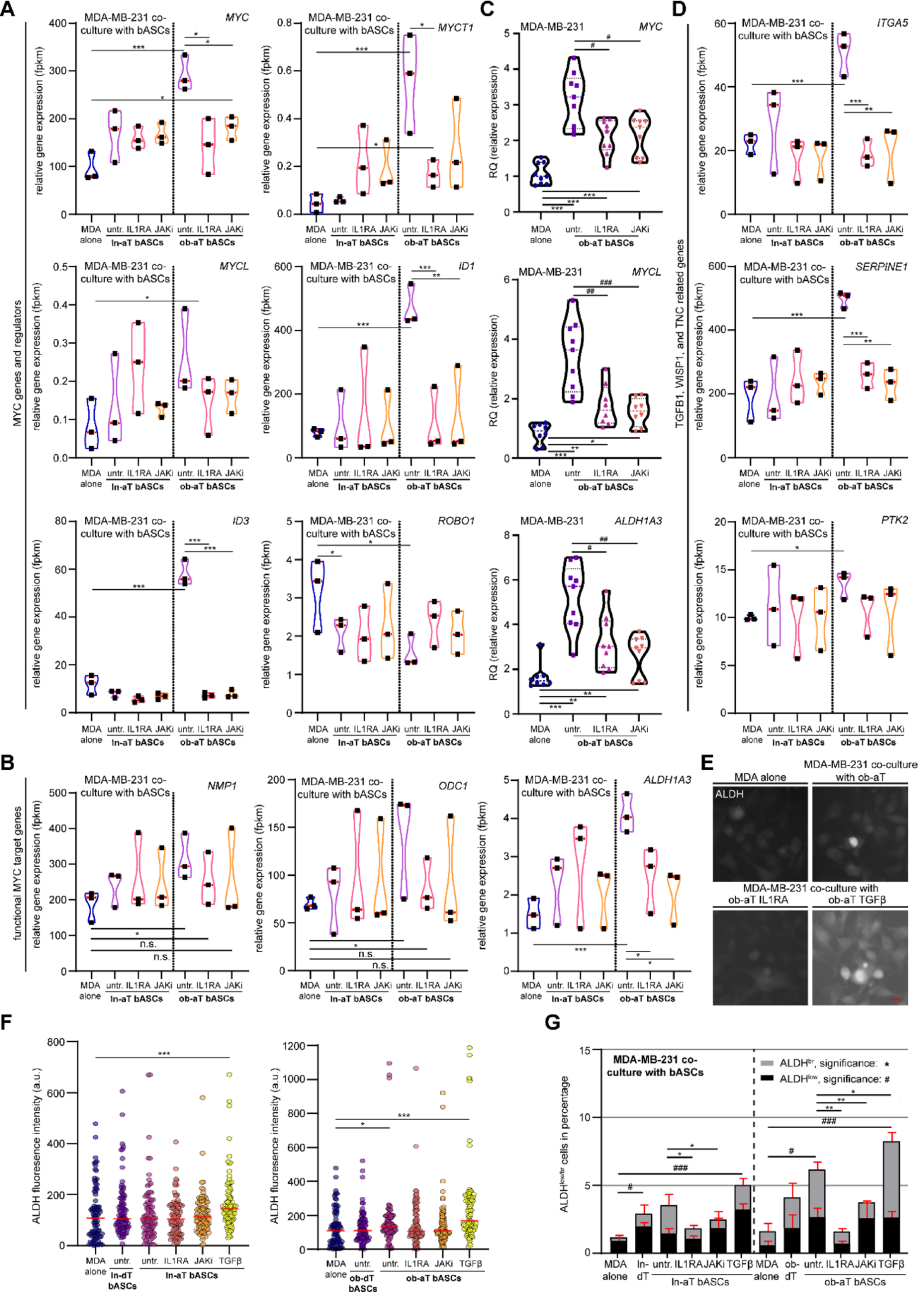
IL1RA or JAKi treated ob-aT bASCs suppress MYC-driven cancer stem cell programs and ALDH activity in co-cultured MDA-MB-231 cells. **A**, **B**, and **D** Violin plots showing RNA-seq expression levels of selected MYC-related transcription factors (*MYC*, *MYCT1*, *MYCL*), positive regulators (*ID1*, *ID3*), negative regulator (*ROBO1*) (**A**), downstream effectors (*NPM1*, *ODC1*,* ALDH1A3*) (**B**), and downstream CSC effectors (*ITGA5*, *SERPINE1*, *PTK2*) (**D**) in MDA-MB-231 cells co-cultured with untreated, IL1RA-treated, JAKi-treated ln- or ob-aT bASCs. **C** Truncated Violin plots display a qPCR analysis of MYC-related genes *MYC*, *MYCL*, and *ALDH1A3* in indicated bASC subgroups. Results are presented as RQ ± SEM (*n* = 3, each includes three replicates). **E**–**G** ALDH activity in MDA-MB-231 cells cultured alone or with indicated bASCs (ln-aT bASCs, ln-aT bASCs treated with IL1RA, ln-aT bASCs treated with JAKi, ln-aT bASCs treated with TGFβ, ob-aT bASCs, ob-aT bASCs treated with IL1RA, ob-aT bASCs treated with JAKi, ob-aT bASCs treated with TGFβ) was assessed by using ALDEFLUOR™ for immunofluorescence staining (**E** and **F**) or flow cytometry (**G**), measuring the percentage of ALDH^br^ and ALDH^low^ cells. Results from three independent experiments are shown as mean ± SEM. **E** Representative images of MDA-MB-231 cells cultured alone or with indicated ob-aT bASC subtypes stained by ALDEFLUOR™. Scale bar, 25 μm. **F** Scatter plot shows quantification of ALDH fluorescence intensity (a.u.) in MDA-MB-231 cells cultured alone or with indicated ln-aT bASC subtypes (scatter plot left) or with indicated ob-aT bASC subtypes (scatter plot right) (*n* = 3, 90 cells measured). **G** Quantification of ALDH^br^ and ALDH^low^ cell populations via flow cytometry (*n* = 3, > 30,000 cells measured). The student’s t-test was used to determine statistical significance. ∗*p* < 0.05, ∗∗*p* < 0.01, ∗∗∗*p* < 0.001

Consistent with the above findings, we observed pronounced activation of the MYC transcriptional network in breast cancer cells exposed to myCAF-like ob-aT bASCs. Specifically, genes encoding *MYC*, its family members *MYCT1* (MYC target 1) and *MYCL* (MYCL proto-oncogene), and positive regulators such as *ID1* (inhibitor of DNA binding 1) and *ID3* were significantly upregulated in MDA-MB-231 cells co-cultured with ob-aT bASCs (Fig. 8A). Simultaneously, negative regulators of MYC, including *ROBO1* (roundabout guidance receptor 1), were downregulated (Fig. 8A). Known MYC effector genes, including *NPM1* (nucleophosmin 1), *ODC1* (ornithine decarboxylase 1), and *ALDH1A3* (aldehyde dehydrogenase 1A3) [67], were similarly elevated in the co-cultured MDA-MB-231 cells (Fig. 8B).

Importantly, pretreatment of ob-aT bASCs with IL1RA or JAKi largely normalized the expression of these MYC-associated genes in MDA-MB-231 cells, with the exception of *ROBO1*, *NPM1*, and *ODC1* (Fig. 8A and B). The upregulation of *MYC*, *MYCL*, and *ALDH1A3* was corroborated by qPCR in MDA-MB-231 cells co-cultured with ob-aT bASCs (Fig. 8C, 1st and 2nd violin plots), but not in MDA-MB-231 cells subjected to ob-aT bASCs pretreated with IL1RA or JAKi (Fig. 8C, 2nd vs. 3rd and 4th violin plots). These findings suggest that MYC pathway activation is a key feature of the CSC phenotype induced by ob-aT bASCs, and that inflammatory blockade can attenuate this program.

In addition to the MYC pathway, we next assessed downstream targets of the identified extrinsic factors. MDA-MB-231 cells co-cultured with untreated ob-aT bASCs showed an upregulation of *ITGA5* (integrin α5), *SERPINE1*, and *PTK2*, which are known targets of the TGFB1, WISP1, and TNC signaling, respectively (Fig. 8D, 1st vs. 5th violin plots) [68–70]. Pretreatment of ob-aT bASCs with IL1RA or JAKi significantly reduced the expression of *ITGA5*, *SERPINE1*, and *PTK2* in co-cultured MDA-MB-231 cells (Fig. 8DD, 5th vs. 6th and 7th violin plots). These results indicate that IL1R/JAK blockade can counteract multiple pro-CSC signaling pathways activated by the myCAF-like stromal niche.

It has been reported that the ALDH activity, particularly ALDH1A1 and ALDH1A3, downstream effectors of the MYC pathway as well as *PTK2*, is a hallmark of CSC formation and closely associated with the CSC phenotype [71]. The kit ALDEFLUOR™ was used for measuring its activity [72]. In fact, MDA-MB-231 co-cultured with either ln-aT bASCs treated with TGFβ or ob-aT bASCs displayed a significant increase in ALDH fluorescence intensity (Fig. 8E and F). Moreover, the population of ALDH bright (ALDH^br^) MDA-MB-231 cells was highly elevated compared to control MDA-MB-231 cells (Fig. 8G). IL1RA and JAKi treatment substantially reduced the amount of ALDH^br^ and ALDH^low^ cells in MDA-MB-231 cells co-cultured with ob-aT bASCs (Fig. 8G, right panel). Similarly, reduction in ALDH activity was also observed in MDA-MB-231 cells co-cultured with ln-aT bASCs treated with IL1RA or JAKi, albeit to a lesser extent (Fig. 8G, left panel). Remarkably, pre-incubation of ob-aT bASCs treated with TGFβ drastically increased the ALDH^br^ population in MDA-MB-231 cells (Fig. 8G, last bar), which is in accordance with previous findings that c-MYC overexpression in MDA-MB-231 cells elevated ALDH^+^ populations by approximately 6% [73]. Together, these findings highlight that myCAF-like ob-aT bASCs drive CSC formation in TNBC cells via activation of MYC- and ALDH-associated programs, and that pharmacologic inhibition of IL1R/JAK1/2 signaling reprograms ob-aT bASCs, an important component of the stromal niche to downregulate multiple CSC pathways and ALDH activity.

## Discussion

In this study, we uncover mechanistic insights into obesity-dependent reprogramming of bASCs within the TME by identifying two distinct stromal phenotypes: an inflammatory iCAF-like and a fibrotic myCAF-like state, both driven by intercellular signals from breast cancer cells and its TME. Our data reveal that bASC identity and function are profoundly shaped by the proximity to cancer tissue and patient metabolic status, with obesity serving as a decisive modulator of bASC fate, transcriptomic profile, and tumor-supportive capacity, thereby substantiating previous findings [25, 74].

Our data have identified a large set of differentially expressed genes in ln- and ob-aT bASCs. Notably, ln-aT bASCs exhibit a significant upregulation of genes involved in multiple pathways, such as TGFβ, NF-κB, MAPK, and Hh, important in the de-differentiation of bASCs into CAF-like phenotypes [56, 75], whereas ob-aT bASCs showed an enriched gene expression in pathways associated with metabolic processes, PI3K-Akt signaling, and ECM remodeling, suggesting a myCAF-like functional phenotype, as described for obesity-associated stromal cells [76, 77]. Coherent with this finding, the data reveal that ln-aT bASCs acquire to a greater extent an inflammatory signature with elevated expression of *LIF*, *CXCL5*, and *IL1B*, increased motility, and secretion of cytokines driving EMT in luminal breast cancer cells [16, 25]. In contrast, ob-aT bASCs adopt a more fibrotic phenotype with strong ECM remodeling capacity, upregulation of *COL1A1*, *FN1*, and *ACTA2*, and high contractility, consistent with previously described obesity-associated stromal reprogramming [17, 76, 78].

A central mechanistic axis uncovered in our study is the TGFβ-driven induction of the myCAF phenotype in obesity-derived bASCs. We demonstrate that TGFβ is both sufficient and necessary to promote myCAF transcriptional programs in ob-dT bASCs, while it has limited impact on ln-dT cells. This may reflect a stromal compartment in obese tissue that is already transdifferentiated toward an activated fibroblast state, rendering it more susceptible to fibrosis-inducing processes. Such transdifferentiation has been shown to be driven by inflammatory cytokines, including IL1, IL6, TNFα, and IFNγ, as well as free fatty acids such as triglycerides and cholesterol [79]. These findings align with prior reports describing TGFβ as a master regulator of fibrosis and stromal remodeling in various malignancies [31, 80]. Notably, scRNA-seq data from Bassez et al. [36] and Nguyen et al. [37] show elevated *TGFB1* expression in macrophages and T cells from obese breast cancer patients, indicating a TME-intrinsic, immune-derived source of this profibrotic signal. Moreover, our findings reveal a compelling similarity between ob-aT bASCs and fibroblasts from NST ER^−^/HER2^−^ breast cancer samples [36, 37], underscoring the obesity-driven reshaping of stromal cells beyond the mesenchymal stem cell compartment. The shared upregulation of ECM components, integrins, and CAF-associated genes highlights the robust transcriptional similarities that define the myCAF-like phenotype in obesity-associated cancers [25, 81].

Importantly, pharmacologic inhibition of IL1 or JAK1/2 signaling using IL1RA or AZD1480 led to a substantial reprogramming of both iCAF- and myCAF-like bASCs, suppressing key inflammatory mediators (*LIF*, *CCL2*, *CXCL1*/2/3) and fibrotic markers (*COL4A1*, *TAGLN*, *FAP*) [29–31, 56]. This expands the findings from colorectal and pancreatic cancer models, where IL1R1^+^ fibroblasts were shown to drive immunosuppression and therapy resistance [29, 31]. Our study is the first to demonstrate that such inhibitors can be applied to human primary bASCs to suppress their tumor-supportive functions in breast cancer.

Functionally, we demonstrate that reprogrammed ln-aT bASCs significantly reduce EMT induction in epithelial breast cancer MCF7 cells, as evidenced by restored epithelial gene and EMT negative regulator expression (*CDH1*, *SOCS2*), downregulation of mesenchymal markers (*VIM*, *SERPINE1*), and a notable reduction in vimentin protein levels. These findings corroborate and extend prior reports showing that stromal cytokine signaling is a key extrinsic driver of EMT in epithelial breast cancer cells [25, 82]. Mechanistically, inhibition of IL1 or JAK1/2 signaling decreased cytokine secretion in ln-aT bASCs, reversed the transcriptional EMT program, and attenuated morphological transformation, emphasizing the therapeutic potential of disrupting stromal-derived inflammatory signaling [83] and targeting EMT within the tumor microenvironment [84].

Ob-aT bASCs induced robust CSC-like traits in TNBC MDA-MB-231 cells, as shown by a pronounced enrichment of a CSC-related gene expression signature and upregulation of *MYC*, *MYCL*, *ALDH1A3*, and downstream effectors such as *ODC1* and *NPM1*. This aligns with previous findings linking MYC activity to CSC maintenance, metabolic reprogramming, and therapeutic resistance in aggressive breast cancer subtypes [71, 85, 86]. Notably, our transcriptomic profiling identified a distinct panel of stromal-derived CSC-promoting factors, including *IGF1*, *EGF*, *TGFB1*, *WISP1*, *TNC*, and *NRG4*, all of which were markedly upregulated in ob-aT bASCs.

Crucially, IL1RA and JAKi treatments suppressed the expression of these extrinsic pro-CSC mediators in bASCs, as reported [68, 69]. In support, GSEA analysis underscored that the CSC signature was no longer significantly enriched in MDA-MB-231 cells co-cultured with inhibitor-treated ob-aT bASCs. Furthermore, MYC pathway activation was significantly attenuated, as shown by the downregulation of MYC targets and normalization of *ALDH1A3* expression. These effects were functionally validated by a significant reduction in ALDH^bright^ cell populations, a hallmark of CSC enrichment [86–88]. Taken together, these data support the notion that CSC induction is driven by a defined panel of extrinsic stromal signals, which are responsive to IL1 and JAK pathway inhibition. By reducing the expression of pro-stemness cytokines and growth factors, both inhibitors effectively reprogram the tumor-supportive phenotype of myCAF-like bASCs, thereby dismantling the stromal niche required for CSC maintenance. These findings provide strong mechanistic support for targeting stromal signaling networks as a mean to impair CSC plasticity and overcome therapy resistance in triple-negative and obesity-associated breast cancer [66, 67, 85, 88].

In addition, obesity profoundly reshapes both the composition and function of immune cell populations, thereby altering the tumor immune microenvironment (TIME) in breast cancer toward an immunosuppressive, tumor-promoting state [89, 90]. In particular, tumor-associated macrophages (TAMs) are established drivers of tumor progression through secretion of pro-inflammatory cytokines, such as TNFα, IL1β, and IL6, and by fostering cancer stem-like cell formation or inducing EMT, which promotes breast cancer growth, metastasis, and therapy resistance [89, 91, 92]. The recruitment of TAMs can be driven by both cytokines and chemokines, among which CXCL12 has been implicated in promoting TAM infiltration in breast cancer [93]. In line with this, the scRNA-seq data reveal a significant upregulation of CXCL12 within the stromal compartment, suggesting a mechanism that may enhance TAM accumulation in the TIME of obese patients. Likewise, cancer-associated adipocytes (CAAs) contribute to tumor aggressiveness by remodeling the ECM and releasing metabolites, cytokines, adipokines, hormones, growth factors, and exosomes, thereby modulating breast cancer cell metabolism, proliferation, migration, invasion, and therapeutic response [94, 95]. Interestingly, several features of aT bASCs in our study mirror hallmarks of CAAs described in breast cancer [94]. Like CAAs, ob-aT bASCs display a fibroblast-like, ECM-remodeling phenotype enriched for myCAF markers and strong contractile capacity, while ln-aT bASCs resemble iCAF-like CAAs through elevated secretion of inflammatory cytokines, such as CCL2 and LIF [76, 94, 95]. This parallel suggests that cancer-educated bASCs and CAAs share core functional programs, namely myofibroblastic ECM remodeling and inflammatory cytokine signaling, which may synergistically reshape the TME and promote breast cancer progression.

Together with our current findings, these observations support a model in which obesity-associated factors modulate multiple adipose tissue-derived cell types, including bASCs, immune cells, and CAAs. These cell types engage in reciprocal interactions that reinforce a pro-tumorigenic microenvironment. Such cross-talks between stromal and immune compartments likely drive breast cancer progression and contribute to therapy resistance. Future studies will be required to delineate the molecular pathways underlying these interactions in both in vitro and in vivo models, thereby deepening our understanding of the TME and TIME in breast cancer patients with obesity.

In conclusion, this study highlights a previously underestimated stroma-cancer axis in the context of obesity, wherein TGFβ-rich, and inflamed microenvironments polarize bASCs toward a myCAF-like phenotype that promote breast cancer progression and stemness. Importantly, reprogramming the stromal compartment with IL1RA or JAK inhibitors emerges as a clinically feasible strategy to mitigate these tumor-promoting effects, particularly in aggressive subtypes such as TNBC [25, 96]. Given that anakinra has already shown clinical promise in phase I/II trials for rectal (NCT04942626) [97] and colorectal cancer (NCT02090101), our findings further support the therapeutic relevance of IL1- or JAK-targeted interventions. These results provide a strong rationale for combining such strategies with standard radio- or chemotherapy in breast cancer, particularly in obese patients whose stromal compartment exhibits a pro-inflammatory, tumor-promoting phenotype, as previously suggested in the context of targeting MSCs during cancer radiotherapy [57, 98]. Interestingly, the ongoing clinical trial OZM-034 (NCT06710197) is evaluating short-term preoperative IL1 inhibition with anakinra in early-stage breast cancer (including TNBC and ER-low disease), with a specific focus on alterations in the TME, including TILs, TAMs, NK cells, and inflammasome activity. For that, our present study provides molecular mechanisms and the translational relevance of IL1-targeted interventions in breast cancer. To further substantiate the efficacy of IL1 or JAK inhibition in stromal reprogramming, future studies utilizing orthotopic in vivo models, patient-derived xenografts, or organoid systems will be essential to translate the mechanistic insights gained in this study with clinically actionable outcomes.

## Supplementary Information

Below is the link to the electronic supplementary material.

Supplementary Material 1.

Supplementary Material 2.

Supplementary Material 3.

## Data Availability

All data generated or analyzed during this study are included in this article and its supplementary information. The scRNA-seq data shown in Fig. 3A and its R code for data analyses are available in a CodeOcean capsule [10.24433/CO.8331460.v1] published by Nguyen et al. [37]. The scRNA-seq dataset supporting the findings of Fig. 3B is available in the Gene Expression Omnibus (GEO) under accession number GSE195665. Patient information for the included samples, along with the BMI-stratified reanalysis, is provided in Supplementary Material 1.
