# Supplementary material for: Obesity- and tumor-derived signals drive cancer-associated state transitions in breast mesenchymal stromal/stem cells reprogrammed by IL1RA or JAK inhibition
Source: Exp Hematol Oncol. 2026 Feb 5;15:16. doi: 10.1186/s40164-026-00747-7 (PMC12882209; doi:10.1186/s40164-026-00747-7)
Supplement: Supplementary file 3 — Supplementary Material 3. [file 40164_2026_747_MOESM3_ESM.docx]

**Online Data Supplement**

**Obesity- and Tumor-Derived Signals Drive Cancer-Associated State Transitions in Breast Mesenchymal Stromal/Stem Cells Reprogrammed by IL1RA or JAK Inhibition**

Andreas Ritter^1^, Samira Catharina Hoock^1^, Nina-Naomi Kreis^1^, Susanne Roth^1^,

Rosario Carolina Torres Colin^1^, Alexandra Friemel^1^, Julia Maria Wildner^1^, Ilona Scherr^1^, Frank Louwen^1^, Christine Solbach^1^ and Juping Yuan^1^

^1^Obstetrics and Prenatal Medicine, Gynaecology and Obstetrics, University Hospital Frankfurt, J. W. Goethe-University, Theodor-Stern-Kai 7, D-60590 Frankfurt, Germany

Running title: Obesity-Induced Stromal Plasticity in Breast Cancer

**Corresponding Authors:**

Dr. Andreas Ritter: Email: aRitter@em.uni-frankfurt.de; Tel.: +49 69 6301 83297.

Obstetrics and Prenatal Medicine, Gynecology and Obstetrics, University Hospital Frankfurt, J. W. Goethe-University, Theodor-Stern-Kai 7, D-60590 Frankfurt, Germany

We declare no conflict of interest, both financial and personal.

**1. Methods**

**1.1. Cell viability, cell cycle distribution and flow cytometry (FACS)**

Cell viability was assessed using the CellTiter-Blue^®^ assay (#G808B, Promega, Walldorf) following the manufacturer’s protocol. For cell cycle analysis, cells were ethanol-fixed (80%, 4°C, 30 min), treated with RNase A (1 mg/ml, #232-646-6; Sigma-Aldrich, Taufkirchen), and stained with propidium iodide (100 μg/ml, #P1304MP, Sigma-Aldrich) at 37°C for 30 min. DNA content was quantified via FACSCalibur™ (BD Biosciences, Heidelberg) and Flowing Software 2.5.1 (Perttu Terho).

Surface marker expression was analyzed by fixing trypsinized cells in 2% PFA (4°C, 15 min), followed by washing twice with FCB buffer (PBS, 0.2% Tween-20, 2% FCS) and staining with following antibodies (eBioscience, Frankfurt am Main): FITC-conjugated anti-human CD90, PerCP-Cy5.5-conjugated anti-human CD90, PerCP-Cy5.5-conjugated anti-human CD14, FITC-conjugated anti-human CD34, PE-conjugated anti-human CD146, PE-conjugated anti-human CD105, APC-conjugated anti-human CD106 and APC-conjugated anti-human CD31. Additionally, following antibodies were purchased from BD Pharmingen^TM^ (Heidelberg): PE-conjugated anti-human CD73, FITC-conjugated anti-human CD14 and APC-conjugated anti-human CD106, PE-conjugated anti-human CD24.

**1.2 Indirect immunofluorescence staining, imaging, and signal intensity measurement**

Cells were seeded on Nunc™ Lab-Tek™ II chamber slides (#C6807-1CS, Thermo Fisher Scientific, Waltham) and fixed in 4% paraformaldehyde supplemented with 0.2% Triton™ X-100 (#T8787, Sigma-Aldrich) for 15 min at room temperature. Primary antibodies employed included: mouse monoclonal anti-vimentin (#M7020, DAKO, Frankfurt), rabbit monoclonal anti-E-cadherin (#3195, Cell Signaling Technology, Leiden), mouse monoclonal anti-fibronectin (#610078, BD Transduction Laboratories^TM^, Darmstadt), mouse monoclonal anti-ENA-78/CXCL5 (D-6) (#sc-377028, Santa Cruz, Heidelberg), rabbit polyclonal anti-COL4A1 (#50273, Cell Signaling Technology), and rabbit monoclonal anti-COL1A1 (#E8I9Z, Cell Signaling Technology). Secondary antibodies conjugated to FITC, Cy3, and Cy5 were obtained from Jackson ImmunoResearch (Cambridgeshire). Nuclear staining was performed using DAPI (Roche, Mannheim), and actin cytoskeleton staining phalloidin-TRITC (#P1951, Sigma-Aldrich).

Microscopy was performed using an AxioObserver Z1 microscope equipped with an AxioCam MRm camera (Zeiss, Oberkochen). Confocal laser scanning microscopy (CLSM, Leica, Wetzlar) was conducted using a Leica CTR 6500 system, employing Z-stack imaging with a HCXPI APO CS 63.0×/1.4 oil-immersion objective. Z-stacks were acquired at 0.5 µm intervals. Representative images were generated by overlaying individual confocal Z-sections utilizing maximum intensity projection (MIP).

Fluorescence intensity was quantified by measuring defined regions of interest (ROI) in ImageJ (v1.5.2i; National Institutes of Health). Mean fluorescence intensities were subtracted against the background intensities and each experimental group was plotted using GraphPad Prism 9 (GraphPad Software Inc., San Diego).

**1.3 RNA extraction, real-time quantitative PCR (qPCR), and transcriptomic analysis (RNA-seq)**

Total RNA was extracted from bASCs, MCF7, and MDA-MB-231 cells using the EXTRACT^ME^ Total RNA Kit (7Bioscience GmbH, Neuenburg am Rhein). Reverse transcription was carried out using the GoScript™ Reverse Transcription Mix with random primers (Promega GmbH, Walldorf). qPCR was performed using a Quantstudio Real-Time PCR System (Applied Biosystems, Darmstadt) with data analyzed via QuantStudio™ v15.2 (Applied Biosystems). Gene expression was quantified using the comparative CT method, represented as ΔCT values normalized to GAPDH as an internal control. Relative quantification (RQ) was calculated as 2^−(ΔCTgroup−ΔCTcontrol)^, where ΔΔCT corresponds to the difference between the ΔCT of the experimental and control groups. Expression levels were reported relative to the control condition (set as 1) with associated minimum and maximum ranges. All TaqMan probes for gene analysis were obtained from Applied Biosystems: *FN1* (#Hs01549976_m1), *COL1A1* (#Hs00164004_m1), *COL4A1* (#Hs00266237_m1), *FAP* (#Hs00990791_m1), *CCL2* (#Hs00234140_m1), *LIF* (#Hs01055668_m1), *IL1R1* (#Hs00491010_m1), *PDGFRB* (#Hs01019589_m1), *PDPN* (#Hs00366766_m1), *ACTA2* (Hs00426835_g1) *MYC* (#Hs00153408_m1), *MYCL* (#Hs00420495_m1), and *ALDH1A3* (#Hs00167476_m1).

For transcriptomic profiling (RNA-seq), total RNA was extracted from bASCs derived from five individual donors with the following bASCs subgroups: ln-aT bASCs, ln-dt bASCs, ob-aT bASCs, and ob-dT bASCs, bASCs non-treated or treated with IL1RA, JAKi, or TGFβ (n = 5 in each subgroup), or from MCF7/MDA-MB-231 cells co-cultured with bASCs pre-treated as indicated for up to 14 days (n = 3 in each subgroup). To further support the hypothesis of BMI-dependent de-differentiation, the RNA-seq data were integrated with our previously published RNA-seq datasets [1], as presented in Fig. 1A and B, Fig. 4D-F, and Fig. S1A and B. The co-cultured cancer cells were sorted via fluorescence-activated cell sorting (FACS) based on negative surface receptors CD90, or CD73, and positive surface marker CD24. For RNA sequencing, 1 μg of RNA per sample was sent to Novogene Co., Ltd. (Munich) for library preparation using the NEBNext® Ultra™ RNA Library Prep Kit for Illumina^®^ (NEB). Differential expression analysis was conducted with DESeq2 (R package) on a dataset encompassing 60,450 genes, with five biological replicates per condition. Genes identified by DESeq2 were further analyzed through Gene Ontology (GO) enrichment and KEGG pathway analysis.

Visualization of differential gene expressions, including heatmaps, volcano plots, violin plots, and pathway analyses, was performed using data provided by Novogene or re-analyzed with Microsoft^®^ Excel^®^ (Redmond, WA) and GraphPad Prism 10 (GraphPad Software Inc.).

Gene set enrichment analysis (GSEA) was performed using version 4.3.3 of the GSEA software (Broad Institute, Massachusetts Institute of Technology, and Regents of the University of California). The epithelial-mesenchymal transition (EMT) gene signature included five genes (*BAMBI*, *CXCR4*, *CDH1*, *EPHA3*, and *SOCS2*) known to be negatively correlated with the phenotype. For these negatively correlated genes, fragments per kilobase per million mapped fragments (fpkm) values were multiplied by −1 to accurately represent negative enrichment in the resulting GSEA plots.

**1.4** **Enzyme-linked immunosorbent assay (ELISA)**

Cells were seeded into 6-cm culture dishes at a density of 360,000 cells per dish and pre-treated with either IL1RA (anakinra, 10 µg/ml), JAK1/2 inhibitor (AZD1480, 500 nM), or TGFβ (5 ng/ml) for 72 h. Cells were then subjected to serum starvation for 72 h. The supernatants were collected for analysis using the following enzyme-linked immunosorbent assays (ELISAs): CCL2 (#DCP00, R&D Systems, Wiesbaden), CXCL1-3 (#ELH-GRO-1, RayBiotech Life, Peachtree Corners), and LIF (#ELH-LIF-1, RayBiotech Life). All assays were performed according to the manufacturers’ protocols.

**1.5 Assessment of the epithelial-to-mesenchymal transition (EMT)**

EMT was induced in MCF7 cells by indirect co-culture with ln-aT bASCs or ob-aT bASCs, which were pre-treated with DMSO, IL1RA, or JAKi for 72 h, in a transwell system for up to 14 days. bASCs were seeded on the upper 6-well culture insert (35.000 cells/well) and MCF7 cells (25.000 cells/well) were cultured on the bottom of 6-well dishes. The medium contained 15% bASC medium and 85% MCF7 medium. The cells were stained for E-cadherin, vimentin, and DNA (DAPI) for immunofluorescence intensity quantification. The percentage of vimentin-positive cells, immunofluorescence intensities of both proteins, and the cell size were analyzed using ImageJ 1.49i software (National Institutes of Health). The fluorescence background was measured in each individual image and was subtracted from the measured values.

**1.6 Motility and cell attraction assay**

For motility assay, bASCs (ln/ob-dT/aT) cells were seeded in 24-well plates with 35% confluence and imaged at 5-min intervals for 13 h. All time-lapse imaging was performed with an AxioObserver.Z1 microscope (Zeiss), imaged with an AxioCam MRc camera (Zeiss) equipped with an environmental chamber to maintain proper environmental conditions (37°C, 5% CO_2_). The time-lapse movies were analyzed by using ImageJ 1.49i software (National Institutes of Health) with the manual tracking plugin, and Chemotaxis and Migration Tool (Ibidi GmbH, Gräfelfing). Tracks were derived from raw data points and were plotted in GraphPad Prism 9 (GraphPad software Inc.). The accumulated distance was calculated using the raw data points via the Chemotaxis and Migration Tool. Thirty random cells per experiment were analyzed and the experiments were repeated independently three times. The patterns of motility were evaluated as described [2].

Cell attraction homing assays were conducted using culture inserts with a 500 µm cell-free gap (#80209, Ibidi GmbH). Inserts were placed in 6-cm culture dishes, and both wells of each insert were seeded with MDA-MB-231 cells (6.5 × 10⁴) and bASCs (5.5 × 10⁴). After a minimum incubation period of 8 h, inserts were carefully removed, and the medium was replaced with bASC medium under the appropriate experimental conditions. Bright-field images were acquired at 12- and 16 h post-insert removal. For each insert, five images were captured, and experiments were performed in duplicate for at least three independent times. For analysis, cells were fixed and stained to visualize the actin cytoskeleton (phalloidin), focal adhesion protein paxillin, centrosome marker pericentrin, and DNA. The length of cell protrusions was quantified by measuring the distance between the pericentrin signal at the centrosome and the paxillin signal at the tip of the protrusion using immunofluorescence images. Measurements were conducted using ImageJ software (v1.49i; National Institutes of Health).

### **1.7 Key resources table**

| **REAGENT or RESOURCE** | **SOURCE** | **IDENTIFIER (Cat #; RRID)** |
| --- | --- | --- |
| **Antibodies** | | |
| Anti‑E‑Cadherin Mouse mAb | BD Biosciences | Cat#610181; RRID: AB_397581 |
| Anti‑Vimentin (D21H3) XP® Rabbit mAb | Cell Signaling Technology | Cat #5741; RRID: AB_10695739 |
| Alexa Fluor® 488 Phalloidin | Cell Signaling Technology | Cat #8878 |
| Anti‑Paxillin (E6R6Z) Rabbit mAb | Cell Signaling Technology | Cat #50195; RRID: AB_2492524 |
| Anti‑Pericentrin Rabbit mAb | Abcam | Cat #ab4448; RRID: AB_298112 |
| DAPI staining solution | Thermo Fisher Scientific | Cat #H1398 — |
| Anti‑COL1A1 (E8F4L) XP® Rabbit mAb | Cell Signaling Technology | Cat #72026; RRID: AB_2799491 |
| Anti‑COL4A1 (E9T4L) Rabbit mAb | Cell Signaling Technology | Cat #75087; RRID: AB_2800224 |
| Anti‑Fibronectin (FN1) Mouse mAb | **Abcam** | ab6328; RRID: AB_305635 |
| Anti-ENA-78 (CXCL5) (D-6) Mouse mAb | **Santa Cruz Biotechnology, Inc.** | Cat #sc-377026 |
| **Chemicals, peptides, and recombinant proteins** | | |
| IL-1 Receptor Antagonist (Anakinra / IL1RA) | Sobi (Germany import, Biozol) | **KINERET®** (Anakinra), EU Import Cat #143090‑92‑0 (CAS) |
| JAK1/2 Inhibitor (AZD1480) | SelleckChem | Cat #S2162; Purity 99.16 % |
| Recombinant Human TGF‑β1 | R&D Systems | Cat #240‑B‑002/CF |
| Recombinant Human IL‑1α | Thermo Fisher | Cat #PHC0014 |
| Recombinant Human IL‑1α | Carl Roth | Cat #3EPX.2 (Fc‑Tag) |
| Recombinant Human LIF | Cell Signaling Tech. | Cat #62226 |
| **Critical commercial assays** | | |
| ALDEFLUOR™ Kit | STEMCELL Technologies | Cat #01700 |
| Culture‑Insert 2 Well (μ‑Dish) | ibidi GmbH | Cat #80209 |
| **Deposited data Repository & Accession** | | |
| scRNA‑seq (and scTCR/CITE‑seq) from early breast cancer patients (BioKey cohort, Bassez et al.) | European Genome–Phenome Archive (EGA):**EGAS00**0**01004809**, **EGAD00001006608** |  |
| **Experimental models: Cell lines** | | |
| MCF7 | ATCC | Cat#HTB-22 |
| MDA-MB-231 | ATCC | Cat#HTB-26 |
| Primary bASCs (ln-aT, ob-aT, ln-dT, ob-dT) | N/A | N/A |
| **Software and algorithms** | | |
| FIJI | NIH | https://imagej.net/software/fiji/downloads |
| Prism | GraphPad | https://www.graphpad.com/features |
| R | The R Project | Version 4.4.2; <https://www.r-project.org/> |
| DESeq2 | \| Bioconductor \| \| --- \| | v1.40.2 (Bioc 3.18) |
| clusterProfiler | Bioconductor | v4.9.3 (Bioc 3.21) |
| FlowJo | FlowJo LLC (BD) | v10.10 (latest stable; released 2023‑11‑16) |
| AxioVision | Carl Zeiss Microscopy GmbH | Version 4.8.2.3 (latest 2024) |
| Leica LAS X | Leica Microsystems | Version 5.3.0 (released 2025‑01‑20) |
| MotilityTracker | GPL‑licensed software | Version 1.5.8; https://sourceforge.net/projects/motilitytracker |

**2. References**

1. Ritter A, Kreis NN, Roth S, Friemel A, Safdar BK, Hoock SC, Wildner JM, Allert R, Louwen F, Solbach C *et al*: **Cancer-educated mammary adipose tissue-derived stromal/stem cells in obesity and breast cancer: spatial regulation and function**. *Journal of experimental & clinical cancer research : CR* 2023, **42**(1):35.

2. Ritter A, Friemel A, Kreis NN, Louwen F, Yuan J: **Impact of Polo-like kinase 1 inhibitors on human adipose tissue-derived mesenchymal stem cells**. *Oncotarget* 2016, **7**(51):84271-84285.

**3. Supplemental tables**

**Supplementary table 1:** Clinical information of breast cancer patients.

|  | Pat. no. | BC type | Grade | Cancer | Nodes | Metas-  tasis. | Receptors | KI67 | Pretreated | Other diseases |
| --- | --- | --- | --- | --- | --- | --- | --- | --- | --- | --- |
| Lean | 13 | 7 NST,  5 inv. lob.,  1 inv. duct. | 2 G1  9 G2  2 G3 | 8 T1  4 T2  1 T4b | 10 N0  3 N1 | 4 MX  9 M0 | 7 ER^+^,  4 ER^+^PR^+^,  2 trip. neg. | 1 5%  7 15-20%  4 30%  1 90% | None | 1 T1 DM  2 A. HTN  1 MS  1 HT  1 T2 DM |
| Obese | 11 | 8 NST,  3 inv. lob. | 4 G1  7 G2 | 9 T1  2 T2 | 11 N0 | 5 MX  6 M0 | 4 ER^+^,  7 ER^+^PR^+^ | 2 5%  6 10-15%  2 20-25%  1 35-45% | 1 TMX  1 RTx | 2 A. HTN  2 HTN  1 T2 DM  1 PA |

Abbreviation: Pat. no: patient numbers; KI67: proliferation marker Ki-67, NST: no special type; inv. lob.: invasive lobular; inv. duct.: invasive ductal; G: Grade, ER: estrogen receptor; PR: progesterone receptor; trip. neg.: triple negative; TMX: tamoxifen; RTx: radiotherapy; T1/T2 DM: type 1/2 diabetes mellitus; A. HTN.: arterial hypertension; MS: multiple sclerosis; HT, hypothyroidism; PA, polyarthritis.

| **Supplementary table 2:** Cell surface markers of lean bASCs. | | | | | | |
| --- | --- | --- | --- | --- | --- | --- |
|  | | | | | | |
| **bASC No.:** | **CD90** | **CD73** | **CD146** | **CD14** | **CD31** | **CD106** |
| ln-aT I | 92.47 | 97.64 | 37.54 | 0.22 | 0.48 | 1.4 |
| ln-dT I | 77.11 | 96.73 | 46.79 | 0.1 | 8.42 | 0.5 |
| ln-aT II | 86.33 | 99.92 | 11.88 | 0 | 6.02 | 1.1 |
| ln-dT II | 90.09 | 99.97 | 24.8 | 2.128 | 0.1 | 11.82 |
| ln-aT III | 87.54 | 87.74 | 12.66 | 0.2 | 6.86 | 0.03 |
| ln-dT III | 73.86 | 80.97 | 0.05 | 0.02 | 12.84 | 9.64 |
| ln-aT IV | 80.6 | 91.87 | 0.12 | 0.38 | 0.15 | 8.2 |
| ln-dT IV | 98.95 | 85.74 | 0.18 | 0.02 | 9.48 | 5.96 |
| ln-aT V | 95.58 | 98.54 | 38.15 | 0.68 | 0.94 | 0.58 |
| ln-dT V | 89.66 | 94.01 | 84.59 | 3.22 | 0.64 | 0.54 |
| ln-aT VI | 96.55 | 94.38 | 44.31 | 0.44 | 18.7 | 5.13 |
| ln-dT VI | 97.67 | 91.37 | 75.24 | 2.6 | 2.02 | 1.86 |
| **ln-aT MV:** | **89.85** | **95.02** | **24.11** | **0.32** | **5.53** | **2.74** |
| **ln-dT MV:** | **87.89** | **91.47** | **38.61** | **1.35** | **5.58** | **5.05** |
| Abbreviation: CD, cluster of differentiation; MV, mean value. | | | | | | |

| **Supplementary table 3:** Cell surface markers of obese bASCs. | | | | | | |
| --- | --- | --- | --- | --- | --- | --- |
|  | | | | | | |
| **bASC No.:** | **CD90** | **CD73** | **CD146** | **CD14** | **CD31** | **CD106** |
| ob-aT I | 63.83 | 95.24 | 37.35 | 0.00 | 4.30 | 3.22 |
| ob-dT I | 92.06 | 96.31 | 15,14 | 0.00 | 5.11 | 1.31 |
| ob-aT II | 91.02 | 99.67 | 93.70 | 1.43 | 2.17 | 2.60 |
| ob-dT II | 87.14 | 99.55 | 98.22 | 7.62 | 0.73 | 3.90 |
| ob-aT III | 69.40 | 84.42 | 0.43 | 0.01 | 12.82 | 0.50 |
| ob-dT III | 91.08 | 88.37 | 1.56 | 0.02 | 2.94 | 0.04 |
| ob-aT IV | 71.44 | 98.78 | 33.83 | 2.45 | 0.16 | 0.10 |
| ob-dT IV | 98.64 | 96.27 | 95.44 | 0.58 | 3.11 | 1.01 |
| ob-aT V | 99.06 | 99.31 | 94.66 | 0.81 | 0.21 | 0.44 |
| ob-dT V | 96.39 | 92.04 | 18.33 | 2.57 | 2.50 | 2.14 |
| ob-aT VI | 97.67 | 91.37 | 75.24 | 1.47 | 2.60 | 5.73 |
| ob-dT VI | 87.44 | 91.58 | 93.28 | 1.94 | 2.54 | 2.09 |
| **ob-aT MV:** | **82.07** | **94.80** | **55.87** | **1.03** | **3.71** | **2.10** |
| **ob-dT MV:** | **92.13** | **94.02** | **61.37** | **2.12** | **2.82** | **1.75** |
| Abbreviation: CD, cluster of differentiation: MV, mean value. | | | | | | |

**4. Supplementary figure legends**

**
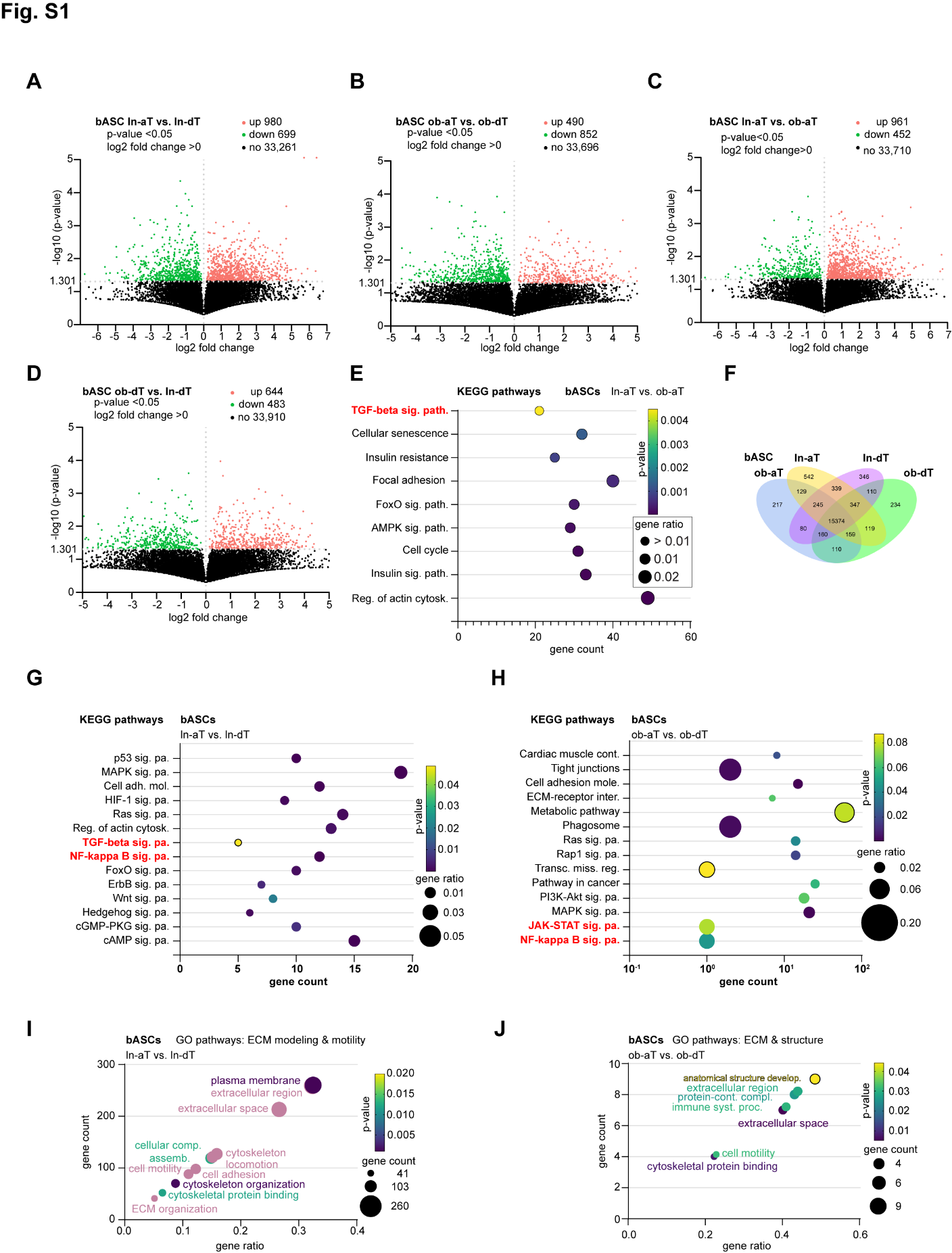
**

**Figure S1: Transcriptomic analysis of bASCs highlights differential gene expression and pathway enrichment associated with proximity to breast cancer and patient metabolic status.**

(**A-J)** Total RNAs were extracted from each sample of bASCs from adipose tissue adjacent (aT) or distant to breast cancer (dT) from patients with normal weight (lean, BMI < 25) or obesity (ob, BMI ≥ 35) (four subgroups: ln-dT, ln-aT, ob-dT and ob-aT, and 5 samples for each subgroup) for transcriptome analysis. (A-D) Volcano plots showing differentially expressed genes (DEGs) between bASCs isolated from tumor-adjacent (aT) and tumor-distant (dT) adipose tissue of breast cancer patients with normal weight (ln, A) or obesity (ob, B), as well as DEGs comparing lean versus obese bASCs in tumor-adjacent (ln-aT vs. ob-aT; C) and tumor-distant (ln-dT vs. ob-dT; D) conditions. DEGs are visualized with adjusted *p*-values (adj. *p* ≤ 0.05) on the y-axis and log2 fold change on the x-axis. Upregulated genes are marked in red, downregulated genes in green, and non-significant genes in black. Analysis was performed using the DESeq2 R package. (**E**) Kyoto encyclopedia of genes and genomes (KEGG) pathway enrichment analysis of DEGs between bASCs from lean and obese tumor-adjacent adipose tissue (ln-aT vs. ob-aT). Pathways are ranked by significance, with p-values indicated by the color gradient and gene ratio (proportion of DEGs in each pathway) represented by dot size. (**F**) Venn diagram showing the distribution of unique and shared significant DEGs (*p* ≤ 0.05) in four bASC subgroups: ln-dT, ln-aT, ob-dT, and ob-aT. Overlapping regions represent common transcriptional changes among subgroups. (**G and H**) KEGG pathway enrichment analysis of DEGs in aT bASCs compared dT bASCs, shown separately for lean (G: ln-aT vs. ln-dT) and obese subgroups (H: ob-aT vs. ob-dT). Pathways are ranked by significance, with *p*-values indicated by the color gradient and gene ratio (proportion of DEGs in each pathway) represented by dot size. Pathways marked in red are particularly relevant for bASC de-differentiation. (**I and J**) Gene Ontology (GO) enrichment analysis for biological processes associated with DEGs in lean (I: ln-aT vs. ln-dT) and obese (J: ob-aT vs. ob-dT) bASCs. The y-axis indicates the number of deregulated genes within each pathway, while the x-axis represents the gene ratio. Color coding reflects the statistical significance of pathway enrichment, with larger dots denoting more gene counts.


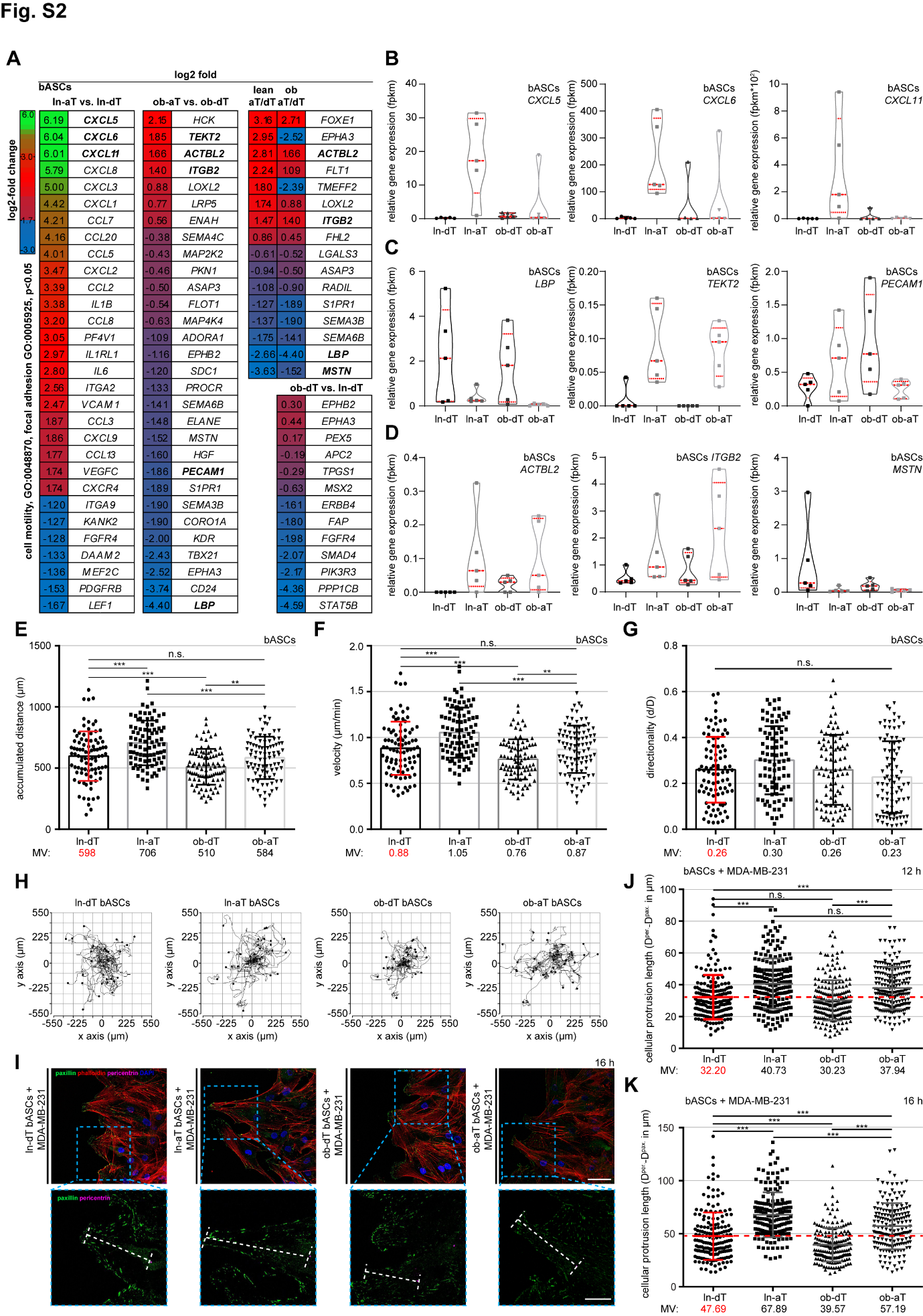


**Figure S2: Divergent molecular mechanisms enhance the motility of ln- and ob-aT bASCs.**

(**A-D)** RNA-seq analysis of different bASCs subgroups (ln-dT, ln-aT, ob-dT and ob-aT, 5 samples for each subgroup). Heatmaps illustrating DEGs in cell motility and focal adhesion pathways (GO: 0048870, 0005925) in bASC subgroups. Comparisons include ln-aT vs. ln-dT (1^st^ panel), ob-aT vs. ob-dT (2^nd^ panel), shared DEGs between ln-aT/dT and ob-aT/dT (3^rd^ panel), and ob-dT vs. ln-dT (4^th^ panel). Log2-fold changes are represented by color intensity (green: +6; red: +3; dark blue: -3). (**B-D**) Violin plots showing relative expression levels (fpkm) of selected DEGs related to cell motility/focal adhesion: DEGs in ln-aT bASCs (B), DEGs in ob-aT bASCs (C), and DEGs shared between ln-aT/dT and ob-aT/dT subgroups (D). (**E-H**) Analysis of bASC single cell motility tracked over 13 h using time-lapse microscopy. Scatter plots represent accumulated distance (E, left), velocity (F, middle), and directionality (G, right) for each bASC subgroup. Data are presented as mean ± SEM (n = 3, 90 cells in each experiment). Representative trajectories of individual cells are shown (H, n = 3, 30 cells per group in each experiment). (**I-K**) Attraction homing assay assessing bASC movement toward MDA-MB-231 cells. After 6 h, Ibidi chambers were removed, allowing bASCs to migrate toward breast cancer cells. At 12 and 16 h, cells were stained for actin filaments (phalloidin, red), focal adhesion (paxillin, green), centrosomes (pericentrin, pink), and DNA (DAPI, blue). Representative images of bASCs at the migratory front at 16 h are shown (I). Scale bar: 50 μm. Scatter plots present the length of cellular protrusions, defined as the distance between pericentrin and paxillin (D^per.^-D^pax.^), at 12 h (J) and 16 h (K). Data represent mean ± SEM (n = 3, 90 protrusions pooled from three independent experiments). Unpaired Mann-Whitney U test was used in (E-G, J, and K). ∗*p* < 0.05, ∗∗*p* < 0.01, ∗∗∗*p* < 0.001.

**
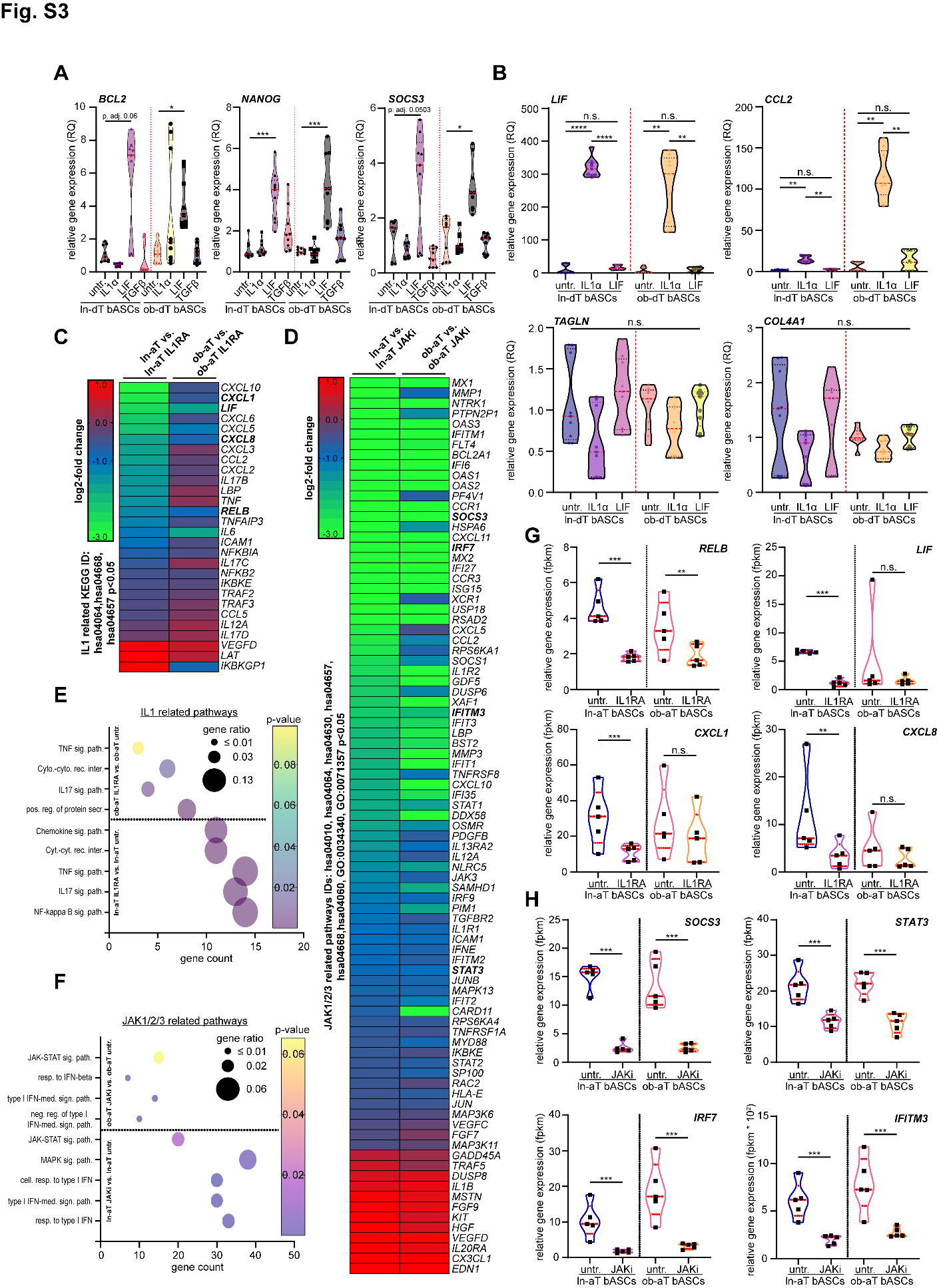
**

**Figure S3: IL1RA and JAKi target their respective pathways in lean and obese bASCs**

**(A and B**) Violin plots depicting the relative expression levels (RQ) of selected LIF downstream targets (*BCL2*, *SOCS3*, *NANOG*) and CAF subtype-related genes (*LIF*, *CCL2*, *TAGLN*, *COL4A1*) in ln- and ob-dT bASCs under different treatment conditions (untreated, IL1α: 5 ng/ml, LIF: 150 ng/ml, 3 days). (**C-H**) RNA-seq analysis of ln- and ob-aT bASCs treated with DMSO, IL1RA anakinra or JAKi AZD1480 as indicated. Heatmaps illustrating DEGs in IL1-related pathways (hsa04064 and hsa04668) (C) or JAK1/2/3 related pathways (KEGG: hsa04010, hsa04064, hsa04630, hsa04657, has04668, hsa04060, GO: 0034340, 0071357) (D) of treated bASCs. Log2-fold changes are represented by color intensity (red: +1; dark blue: -1; green: -3). KEGG pathway enrichment bubble plot of IL1-related pathways in (E) and JAK1/2/3 related pathways in (F). Pathways are ranked by significance, with p-values indicated by the color gradient. X-axis depicts the gene count of each pathway and the gene ratio (proportion of DEGs in each pathway) is presented by dot size. Violin plots showing relative expression levels (fpkm) of selected DEGs associated with the IL1 (G) or JAK1/2/3 (H) pathways in bASCs subgroups and conditions as indicated.

**
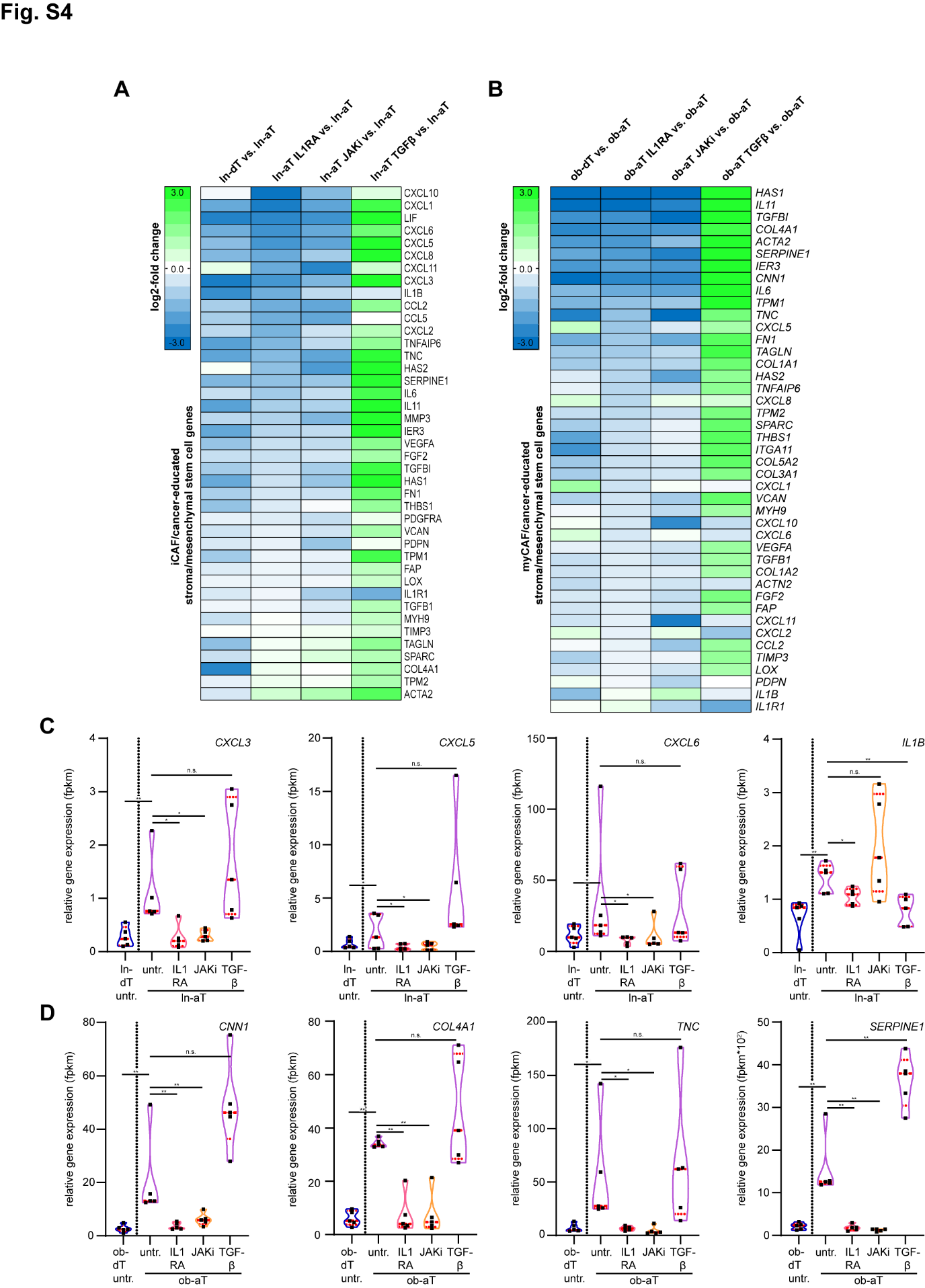
**

**Figure S4: IL1RA and JAKi suppress key iCAF and myCAF gene expression profiles in bASCs**

RNA-seq analysis of ln- and ob-aT bASC subgroups treated with DMSO, TGFβ, IL1RA anakinra or JAKi AZD1480. (**A and B**) Heatmaps illustrate DEGs associated with iCAF genes (A) or myCAF genes (B) of bASCs treated as indicated. Log2-fold changes are represented by color intensity (green: +3; white: ±0; dark blue: -3). (**C and D**) Violin plots showing relative expression levels (fpkm) of selected DEGs associated with the iCAF phenotype (C) or myCAF phenotype (D) in bASCs treated as indicated.

**
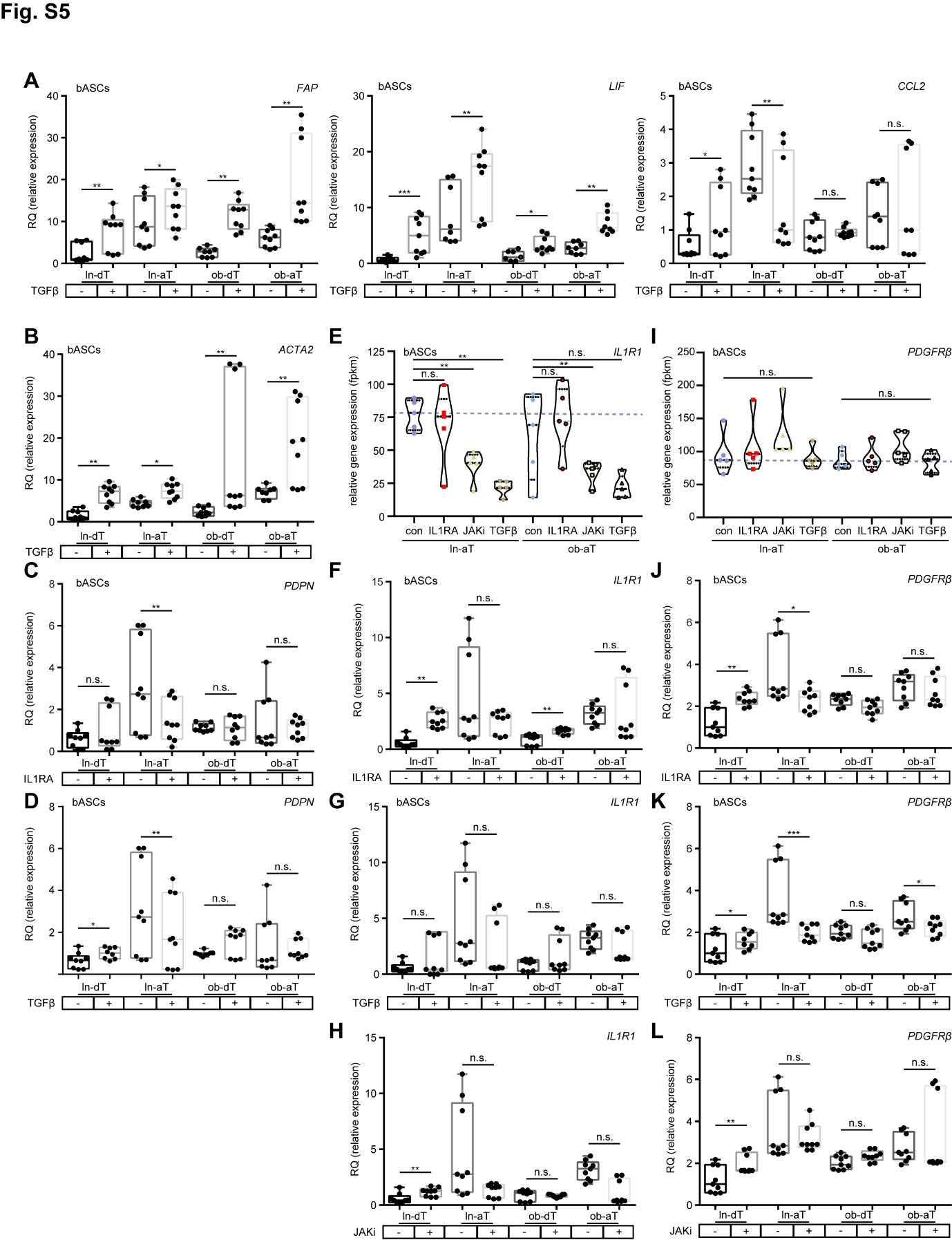
**

**Figure S5: IL1RA, JAKi, and TGFβ have a moderate effect on the gene expression of IL1R1, PDPN, and PDGFRβ in bASCs**

(**A-D**) Quantitative PCR data for CAF-related genes depicting RQ ± SEM values. Dot-boxplots display relative expression levels for *FAP* (A), and *ACTA2* (B) of bASCs treated with TGFβ. Dot-boxplots indicate *PDPN* gene expression in bASCs treated with IL1RA (C), and *PDPN*, *LIF*, and *CCL2* expression in TGFβ treated cells (D). (**E and I)** RNA-seq analysis of ln- and ob-aT bASCs treated with DMSO (con), IL1RA anakinra, JAKi AZD1480, and TGFβ. Violin plots represent RNA-seq-derived relative expression levels (fpkm) of *IL1R1* (E) and *PDGFRβ* (I) in bASC subgroups treated as indicated. (**F**-**H and J-L**) Violin plots display relative gene expression levels of *IL1R1* (F-H) or *PDGFRβ* (J-L) in bASCs subgroups treated with *IL1RA* (F and J), *TGFβ* (G and K), and JAKi (H and L). Student’s t-test was used in (A, B, D-F, H-J). ∗*p* < 0.05, ∗∗*p* < 0.01, ∗∗∗*p* < 0.001.

**
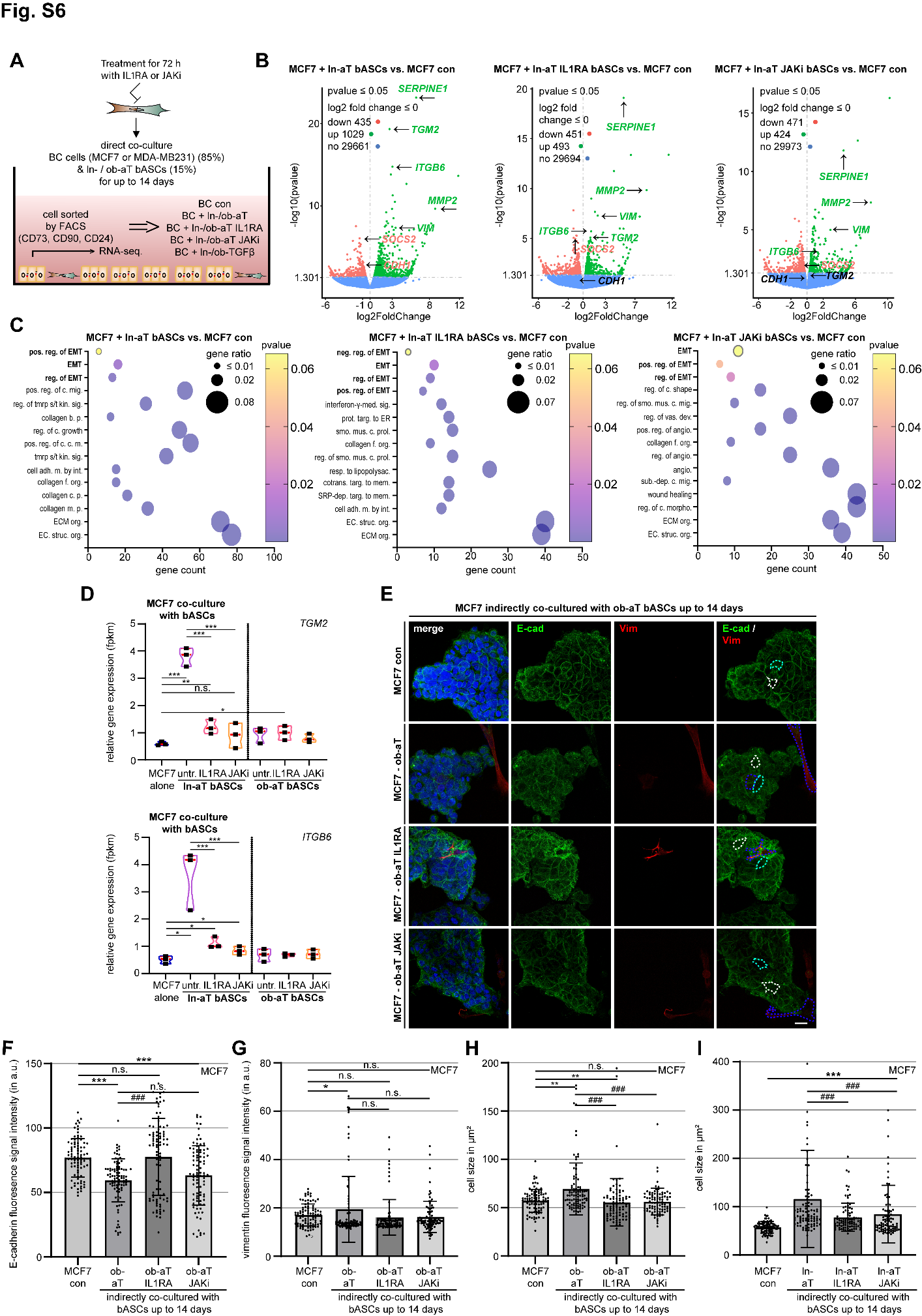
**

**Figure S6: IL1RA or JAKi treated ob-aT bASCs modestly reduce EMT induction in MCF7 cells**

(**A**) Experimental setup: bASCs were pre-treated with DMSO, IL1RA anakinra, or JAKi AZD1480 for 72 h. bASCs and breast cancer cells (MCF7 or MDA-MB-231) were then seeded as a mixed monolayer culture (2D) with direct cell-cell contact for up to 14 days. Cells were then sorted using the cell surface markers CD90/CD73 (bASCs marker), and CD24 (breast cancer cell marker) for further experiments. (**B and C**) RNA-seq analysis of MCF7 cells cultured alone or in direct co-culture with non-treated or treated bASC subgroups (ln-aT bASCs, ln-aT bASCs treated with IL1RA, and ln-aT bASCs treated with JAKi, ob-aT bASCs (n = 3)). The gene expression was analyzed using DESeq2 R package. (B) Volcano plots show DEGs between subgroups: MCF7 + ln-aT bASCs vs. MCF7 con (1^st^), MCF7 + ln-aT bASCs treated with IL1RA vs. MCF7 con (2^nd^), and MCF7 + ln-aT bASCs treated with JAKi vs. MCF7 con (3^rd^). DEGs are visualized with adjusted *p*-values (y-axis) and log2 fold changes (x-axis): upregulated in green, downregulated in red, and non-significant genes in blue. (C) KEGG pathway enrichment bubble plot of indicated conditions. EMT-related pathways are depicted in bold. Pathways are ranked by significance, with *p*-values indicated by the color gradient. X-axis depicts the gene count of each pathway and the gene ratio (proportion of DEGs in each pathway) is represented by dot size. **(D)** Violin plots displaying RNA-seq-derived relative gene expression levels (fpkm) of key mesenchymal genes (*TGM2*, *ITGB6*) in MCF7 cells co-cultured with indicated bASC subgroups. **(E)** Representative images of MCF7 cells alone or directly co-cultured with indicated bASC subgroups. Scale bar, 25 μm. White dotted lines indicate areas used for cell size measurements, blue dotted lines depict areas for vimentin intensity measurements, and cyan lines represent regions for E-cadherin intensity measurements. **(F-I)** Fluorescence intensity quantification of E-cadherin (F), vimentin (G), and cell size of MCF7 cells after co-culture with obese (H) and lean (I) bASCs. Data represent three independent experiments (n = 3, 90 cells per group pooled from three experiments) and are presented as scatter bar plots showing mean ± SEM. Statistical significance was determined using an unpaired Mann-Whitney U test. **p* < 0.05, ***p* < 0.01, ****p* < 0.001.
